# Cortical and subcortical brain structure in generalized anxiety disorder: findings from 28 research sites in the ENIGMA-Anxiety Working Group

**DOI:** 10.1038/s41398-021-01622-1

**Published:** 2021-10-01

**Authors:** Anita Harrewijn, Elise M. Cardinale, Nynke A. Groenewold, Janna Marie Bas-Hoogendam, Moji Aghajani, Kevin Hilbert, Narcis Cardoner, Daniel Porta-Casteràs, Savannah Gosnell, Ramiro Salas, Andrea P. Jackowski, Pedro M. Pan, Giovanni A. Salum, Karina S. Blair, James R. Blair, Mira Z. Hammoud, Mohammed R. Milad, Katie L. Burkhouse, K. Luan Phan, Heidi K. Schroeder, Jeffrey R. Strawn, Katja Beesdo-Baum, Neda Jahanshad, Sophia I. Thomopoulos, Randy Buckner, Jared A. Nielsen, Jordan W. Smoller, Jair C. Soares, Benson Mwangi, Mon-Ju Wu, Giovana B. Zunta-Soares, Michal Assaf, Gretchen J. Diefenbach, Paolo Brambilla, Eleonora Maggioni, David Hofmann, Thomas Straube, Carmen Andreescu, Rachel Berta, Erica Tamburo, Rebecca B. Price, Gisele G. Manfro, Federica Agosta, Elisa Canu, Camilla Cividini, Massimo Filippi, Milutin Kostić, Ana Munjiza Jovanovic, Bianca A. V. Alberton, Brenda Benson, Gabrielle F. Freitag, Courtney A. Filippi, Andrea L. Gold, Ellen Leibenluft, Grace V. Ringlein, Kathryn E. Werwath, Hannah Zwiebel, André Zugman, Hans J. Grabe, Sandra Van der Auwera, Katharina Wittfeld, Henry Völzke, Robin Bülow, Nicholas L. Balderston, Monique Ernst, Christian Grillon, Lilianne R. Mujica-Parodi, Helena van Nieuwenhuizen, Hugo D. Critchley, Elena Makovac, Matteo Mancini, Frances Meeten, Cristina Ottaviani, Tali M. Ball, Gregory A. Fonzo, Martin P. Paulus, Murray B. Stein, Raquel E. Gur, Ruben C. Gur, Antonia N. Kaczkurkin, Bart Larsen, Theodore D. Satterthwaite, Jennifer Harper, Michael Myers, Michael T. Perino, Chad M. Sylvester, Qiongru Yu, Ulrike Lueken, Dick J. Veltman, Paul M. Thompson, Dan J. Stein, Nic J. A. Van der Wee, Anderson M. Winkler, Daniel S. Pine

**Affiliations:** 1 Emotion and Development Branch, National Institute of Mental Health, Bethesda, MD USA; 2 Department of Psychiatry & Neuroscience Institute, University of Cape Town, Cape Town, South Africa; 3 Department of Psychiatry, Leiden University Medical Center, Leiden, The Netherlands; 4 Department of Developmental and Educational Psychology, Institute of Psychology, Leiden University, Leiden, The Netherlands; 5 Leiden Institute for Brain and Cognition, Leiden, The Netherlands; 6 Department of Psychiatry, Amsterdam UMC, location VUMC, Amsterdam, The Netherlands; 7 Department of Research & Innovation, GGZ InGeest, Amsterdam, The Netherlands; 8 Department of Psychology, Humboldt-Universität zu Berlin, Berlin, Germany; 9 Department of Mental Health, University Hospital Parc Taulí-I3PT, Barcelona, Spain; 10 Department of Psychiatry and Forensic Medicine, Universitat Autònoma de Barcelona, Barcelona, Spain; 11 Centro de Investigación Biomédica en Red de Salud Mental, Carlos III Health Institute, Madrid, Spain; 12 Menninger Department of Psychiatry and Behavioral Sciences, Baylor College of Medicine, Houston, TX USA; 13 LiNC, Department of Psychiatry, Federal University of São Paulo, São Paulo, Brazil; 14 Section on Negative Affect and Social Processes, Hospital de Clínicas de Porto Alegre, Universidade Federal do Rio Grande do Sul, Porto Alegre, Brazil; 15 Center for Neurobehavioral Research, Boys Town National Research Hospital, Boys Town, NE USA; 16 Department of Psychiatry, NYU School of Medicine, New York University, New York, NY USA; 17 Department of Psychiatry, University of Illinois at Chicago, Chicago, IL USA; 18 Department of Psychiatry and Behavioral Health, The Ohio State University, Columbus, OH USA; 19 Department of Psychiatry & Behavioral Neuroscience, University of Cincinnati, Cincinnati, OH USA; 20 Behavioral Epidemiology, Institute of Clinical Psychology and Psychotherapy, Technische Universität Dresden, Dresden, Germany; 21 Imaging Genetics Center, Mark and Mary Stevens Neuroimaging and Informatics Institute, Keck School of Medicine, University of Southern California, Marina del Rey, CA USA; 22 Center for Brain Science & Department of Psychology, Harvard University, Cambridge, MA USA; 23 Department of Psychiatry, Massachusetts General Hospital, Boston, MA USA; 24 Psychology Department & Neuroscience Center, Brigham Young University, Provo, USA; 25 Center Of Excellence On Mood Disorders, Louis A. Faillace, MD, Department of Psychiatry and Behavioral Sciences, The University of Texas Health Science Center at Houston, Houston, TX USA; 26 Olin Neuropsychiatry Research Center, Institute of Living, Hartford Hospital, Hartford, CT USA; 27 Department of Psychiatry, Yale School of Medicine, New Haven, CT USA; 28 Anxiety Disorders Center, Institute of Living, Hartford Hospital, Hartford, CT USA; 29 Department of Neurosciences and Mental Health, Fondazione IRCCS Ca’ Granda Ospedale Maggiore Policlinico, Milan, Italy; 30 Department of Pathophysiology and Transplantation, University of Milan, Milan, Italy; 31 Institute of Medical Psychology and Systems Neuroscience, University of Muenster, Muenster, Germany; 32 Department of Psychiatry, University of Pittsburgh, Pittsburgh, PA USA; 33 Department Psychology, University of Pittsburgh, Pittsburgh, PA USA; 34 Anxiety Disorder Program, Hospital de Clínicas de Porto Alegre, Department of Psychiatry, Federal University of Rio Grande do Sul, Porto Alegre, Brazil; 35 Neuroimaging Research Unit, Institute of Experimental Neurology, Division of Neuroscience, IRCCS San Raffaele Scientific Institute, Milan, Italy; 36 Vita-Salute San Raffaele University, Milan, Italy; 37 Neurology Unit, IRCCS San Raffaele Scientific Institute, Milan, Italy; 38 Neurophysiology Unit, IRCCS San Raffaele Scientific Institute, Milan, Italy; 39 Neurorehabilitation Unit, IRCCS San Raffaele Scientific Institute, Milan, Italy; 40 Institute of Mental Health, University of Belgrade, Belgrade, Serbia; 41 Department of Psychiatry, School of Medicine, University of Belgrade, Belgrade, Serbia; 42 Graduate Program in Electrical and Computer Engineering, Universidade Tecnológica Federal do Paraná, Curitiba, Puerto Rico Brazil; 43 Department of Psychiatry and Human Behavior, Brown University Warren Alpert Medical School, Providence, RI USA; 44 Department of Psychiatry and Psychotherapy, University Medicine Greifswald, Greifswald, Germany; 45 German Center for Neurodegenerative Diseases (DZNE), Site Rostock/Greifswald, Greifswald, Germany; 46 Institute for Community Medicine, University Medicine Greifswald, Greifswald, Germany; 47 Institute for Diagnostic Radiology and Neuroradiology, University Medicine Greifswald, Greifswald, Germany; 48 Center for Neuromodulation in Depression and Stress, University of Pennsylvania, Philadelphia, PA USA; 49 Section on Neurobiology of Fear and Anxiety, National Institute of Mental Health, Bethesda, MD USA; 50 Department of Biomedical Engineering, Stony Brook University, Stony Brook, NY USA; 51 Department of Physics, Stony Brook University, Stony Brook, NY USA; 52 Department of Neuroscience, Brighton and Sussex Medical School, University of Sussex, Brighton, UK; 53 Centre for Neuroimaging Science, Kings College London, London, UK; 54 School of Psychology, University of Sussex, Brighton, UK; 55 Department of Psychology, Sapienza University of Rome, Rome, Italy; 56 IRCCS Santa Lucia Foundation, Rome, Italy; 57 Psychiatry and Behavioral Sciences, Stanford University School of Medicine, Stanford, CA USA; 58 Department of Psychiatry and Behavioral Sciences, The University of Texas at Austin Dell Medical School, Austin, TX USA; 59 Laureate Institute for Brain Research, Tulsa, OK USA; 60 Department of Psychiatry, School of Medicine and Herbert Wertheim School of Public Health, University of California, San Diego, La Jolla, CA USA; 61 Department of Psychiatry, University of Pennsylvania, Philadelphia, PA USA; 62 Department of Psychiatry, Washington University, St. Louis, MO USA; 63 South African Medical Research Council Unit on Risk & Resilience in Mental Disorders, Department of Psychiatry & Neuroscience Institute, University of Cape Town, Cape Town, South Africa

**Keywords:** Neuroscience, Psychology

## Abstract

The goal of this study was to compare brain structure between individuals with generalized anxiety disorder (GAD) and healthy controls. Previous studies have generated inconsistent findings, possibly due to small sample sizes, or clinical/analytic heterogeneity. To address these concerns, we combined data from 28 research sites worldwide through the ENIGMA-Anxiety Working Group, using a single, pre-registered mega-analysis. Structural magnetic resonance imaging data from children and adults (5–90 years) were processed using FreeSurfer. The main analysis included the regional and vertex-wise cortical thickness, cortical surface area, and subcortical volume as dependent variables, and GAD, age, age-squared, sex, and their interactions as independent variables. Nuisance variables included IQ, years of education, medication use, comorbidities, and global brain measures. The main analysis (1020 individuals with GAD and 2999 healthy controls) included random slopes per site and random intercepts per scanner. A secondary analysis (1112 individuals with GAD and 3282 healthy controls) included fixed slopes and random intercepts per scanner with the same variables. The main analysis showed no effect of GAD on brain structure, nor interactions involving GAD, age, or sex. The secondary analysis showed increased volume in the right ventral diencephalon in male individuals with GAD compared to male healthy controls, whereas female individuals with GAD did not differ from female healthy controls. This mega-analysis combining worldwide data showed that differences in brain structure related to GAD are small, possibly reflecting heterogeneity or those structural alterations are not a major component of its pathophysiology.

## Introduction

Research on brain structure in generalized anxiety disorder (GAD) has generated inconsistent findings, possibly due to small sample sizes as well as clinical and analytic heterogeneity. The Enhancing NeuroImaging Genetics through Meta-Analysis (ENIGMA) collaboration addresses these challenges in a range of disorders by pooling neuroimaging data across research sites worldwide [1–5]. Here, we employed the ENIGMA approach to investigate differences between individuals with GAD and healthy controls in indices of brain structure in a report from the ENIGMA-Anxiety Working Group [6]. We conducted a structural magnetic resonance imaging (MRI) mega-analysis^1^ using data from 28 research sites worldwide. The current study compared regional and vertex-wise cortical thickness, cortical surface area, and subcortical volume in individuals with GAD and healthy controls, using methods that accommodate data heterogeneity across the research sites.

GAD is a highly prevalent and impairing anxiety disorder notable for its relationship to multiple forms of psychopathology [7, 8]. Where most anxiety disorders develop in late childhood, the median age of onset of GAD is in adulthood [9]. Like other diagnoses, GAD is characterized by clinical heterogeneity, as individuals with GAD could display many different symptoms profiles. Moreover, most individuals with GAD suffer from at least one other mental disorder, particularly other anxiety disorders, major depressive disorder (MDD), and substance use [7, 8, 10]. Longitudinal and family studies show that genetic risks of GAD overlap in part with those of MDD and other anxiety disorders [11–14]. Most prior structural MRI studies rely on voxel-based morphometry (VBM) and reported altered gray matter volume in a wide variety of brain regions in individuals with GAD compared to healthy controls [15–17]. Some, but not others, showed increased gray matter volume in the amygdala and prefrontal cortex (PFC) as well as decreased gray matter volume in the hippocampus [15–17]. Findings on cortical thickness and the surface area appeared similarly inconsistent [17–20]. One potential explanation for this inconsistency is clinical heterogeneity.

Small sample sizes and analytical heterogeneity across individual studies may also generate inconsistent findings. The ENIGMA collaboration provides a solution to these problems, by facilitating the pooling of neuroimaging data across multiple research sites [5, 21]. This is typically done using meta-analyses, where each participating research site first processes and analyzes their local data through a previously agreed common pipeline [1–3]. While this approach addresses concerns with small sample sizes and analytic heterogeneity, it uses pooled data for each site. This prevents the modeling of covariates (such as comorbid disorders) at the individual subject level. The current study addressed the latter problem through a mega-analysis, which can be more powerful than meta-analyses [22], and which allows modeling of covariates at the subject rather than site-averaged level. The mega-analytic approach is used less frequently, as working with individual participant data creates methodological challenges (e.g., study planning and implementation, international transfer of data, quality control of large amounts of data) and requires more computational resources than site-averaged data [23].

The current study assembled raw structural MRI data from 28 research sites, and conducted a pre-registered data analysis [24]. We compared regional and vertex-wise cortical thickness, cortical surface area, and subcortical volume between individuals with GAD and healthy controls while examining interactions with age and sex. Based on prior studies [15–17], we hypothesized that individuals with GAD would show differences in subcortical volume in the amygdala and hippocampus and in cortical thickness and surface area in the PFC compared to healthy controls. We also expected the association between GAD and structural measures to differ as a function of participant age, but we had no specific hypotheses on the direction of this interaction as previous studies examined the effect of GAD within and not across age groups [19, 20]. The analysis in the present work used a whole-brain approach, while accounting for the multiple tests defined in the pre-registration [24].

## Materials and methods

### Participants

The current study is a pre-registered mega-analysis of structural MRI data that had been collected at 28 research sites and repositories from Brazil, Europe, and the USA [24]. As ENIGMA-GAD is an ongoing collaboration, new research groups are encouraged to join. Some site-specific results have been reported before, including from the National Institute of Mental Health (NIMH) team leading the current project [16, 18, 25–29]. However, no reports have examined results across these and additional samples using a pre-registered plan. Twenty-five ENIGMA-GAD sites sent raw individual participant MRI data. Additionally, raw structural MRI data were downloaded from three publicly available imaging repositories to increase sample size and thus, allow more stable estimates of eventual effects: Adolescent Brain Cognitive Development Study (ABCD) [30, 31], Child Mind Institute Healthy Brain Network (CMI-HBN) [32], and Duke Preschool Anxiety Study [33]. All 25 research sites signed an individual data use agreement with the NIMH that included regulations about data use, subject identification, data transfer methods, data ownership, and confidentiality and security practices [23]. Data use guidelines of the repositories were followed. All adult participants and parents of child participants provided written informed consent at their local research site, and the individual research protocols were approved by local institutional review boards and ethics committees.

Data were included if individuals were diagnosed with current or past GAD^2^, not necessarily as the primary diagnosis. Exclusion criteria for individuals with GAD were current or past autism spectrum disorders, bipolar disorder, psychosis, or schizophrenia. These decisions regarding inclusion and exclusion reflected past results from ENIGMA, where robust differences in morphometry were found in studies of the excluded conditions [1, 4]. Comparison subjects were excluded if they had any current or past mental disorder. Diagnoses were based on standardized interviews with a clinician at each research site (see Bas-Hoogendam et al. (2020) for an overview).

**Fig. 1:**
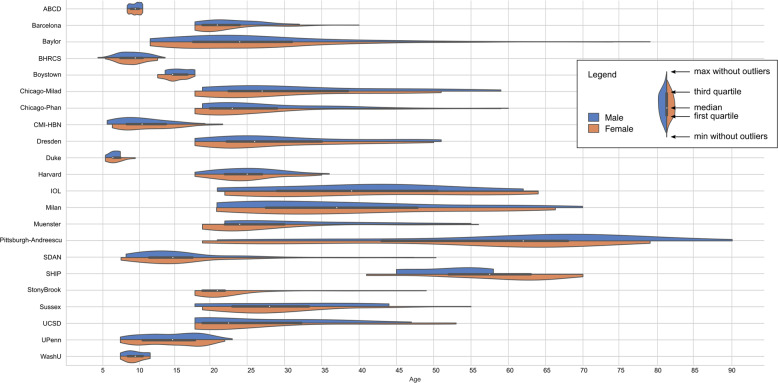
Violin plots of the age distribution for all sites in the main analysis. ABCD Adolescent Brain Cognitive Development Study, BHRCS Brazilian High Risk Cohort Study, CMI-HBN Child Mind Institute Healthy Brain Network, IOL Institute of Living, SDAN Section on Development and Affective Neuroscience, SHIP Study of Health in Pomerania, UCSD University of California – San Diego, UPenn University of Pennsylvania, WashU Washington University.

We received data from 5523 participants before pre-registration [24] (Table 1 shows the number of participants in each step of the analysis). There were some small changes to this number after pre-registration and before pre-processing of the data (see Supplementary Information for the exact numbers and reasons per site for the differences). Table 2 shows the reasons for excluding data. The main pre-registered analysis with random slopes for all independent variables per site and random intercepts per scanner included 1020 individuals with GAD (685 females, *M*_age_ = 23.65 years, SD_age_ = 13.15) and 2999 healthy controls (1617 females, *M*_age_ = 14.76 years, SD_age_ = 10.01), ranging from 5 to 90 years (Fig. 1). Table 3 shows descriptive statistics, Table 4 comorbid diagnoses for individuals with GAD, and Table 5 medication status for participants included in this main analysis. More sites and participants could be included in the secondary analysis with fixed slopes for all independent variables and random intercepts per scanner: 1112 individuals with GAD (753 females) and 3282 healthy controls (1805 females), ranging from 5 to 90 years (*M* = 18.47, SD = 12.72). These additional participants were from sites that had sample sizes that were too small to allow modeling random slopes (see statistical analysis); these sites could only be included with fixed slopes. Supplementary Table 1 shows the descriptive statistics, Supplementary Table 2 the comorbid diagnoses for individuals with GAD, and Supplementary Table 3 medication status for the participants who were included in this secondary analysis. The three imaging repositories consisted of data from multiple scanners: ABCD (29 scanners), CMI-HBN (4 scanners), and Duke (2 scanners). In addition, two sites also contributed data from multiple scanners: Brazilian High Risk Cohort Study (BHRCS; 2 scanners) and Section on Development and Affective Neuroscience (SDAN; 4 scanners).

**Table 1:** Overview of participants in different steps of the analysis.

Site	Location	Pre-registration			Initial number of images			Number of images of high quality			Number of participants included in main analysis with random slopes and random intercepts			Number of participants included in secondary analysis with fixed slopes and random intercepts		
		GAD	HC	Total	GAD	HC	Total	GAD	HC	Total	GAD	HC	Total	GAD	HC	Total
ABCD [30, 31]	USA	114	1495	1609	112	1488	1600	105	1355	1460	104	1347	1451	104	1347	1451
Barcelona [48]	Spain	31	60	91	32	59	91	32	59	91	31	58	89	31	58	89
Baylor [49]	USA	98	0	98	98	130	228	98	130	228	98	130	228	98	130	228
BHRCS [50]	Brazil	101	668	769	19	750	781^a^	15	523	543^a^	15	373	388	15	373	388
Boystown [51, 52]	USA	50	45	95	50	45	95	49	45	94	49	44	93	49	44	93
Chicago-Milad [53]	USA	27	16	43	27	16	43	27	16	43	27	16	43	27	16	43
Chicago-Phan [54, 55]	USA	104	43	147	79	68	147	78	66	144	78	42	120	78	42	120
Cincinnati [56]	USA	10	11	21	9	12	21	3	2	5	Excluded: no HCs			3	0	3
CMI-HBN [32]	USA	121	170	291	120	170	290	102	140	242	39	54	93	39	54	93
Dresden [27]	Germany	47	47	94	47	47	94	47	47	94	47	47	94	47	47	94
Duke [33]	USA	26	19	45	21	24	45	11	14	25	11	12	23	11	12	23
Harvard	USA	203	57	260	142	118	260	123	99	222	122	41	163	122	41	163
Houston [57]	USA	9	264	273	12	261	273	7	203	210	Excluded: all patients had MDD			6	184	190
IOL [58, 59]	USA	43	21	64	33	31	64	25	23	48	24	16	40	24	16	40
Milan [18]	Italy	34	64	98	34	64	98	34	63	97	29	38	67	29	38	67
Muenster [60]	Germany	25	29	54	25	29	54	25	29	54	24	29	53	24	29	53
Pittsburgh-Andreescu [61]	USA	38	41	79	38	41	79	29	34	63	27	32	59	27	32	59
Pittsburgh-Price [62, 63]	USA	69	0	69	69	0	69	69	0	69	Excluded: no HCs			55	0	55
PROTAIA [64, 65]	Brazil	26	18	44	13	31	44	10	28	38	Excluded: rank deficient			4	10	14
SanRaffaele [25]	Italy	21	71	92	21	71	92	21	71	92	Excluded: all patients had MDD and medication			21	70	91
SDAN [29, 66]	USA	243	166	409	168	238	406	152	226	378	148	219	367	148	219	367
SHIP [67]	Germany				12	24	36	12	24	36	12	24	36	10	24	34
SNFA [68]	USA	23	40	63	20	43	63	13	25	38	Excluded: rank deficient			5	19	24
StonyBrook [26]	USA	41	20	61	41	20	61	41	20	61	40	20	60	40	20	60
Sussex [69, 70]	UK	19	21	40	19	21	40	19	21	40	19	21	40	19	21	40
UCSD [71, 72]	USA	46	50	96	46	50	96	45	45	90	45	39	84	45	39	84
UPenn [73, 74]	USA	27	428	455	27	428	455	24	370	394	11	370	381	11	370	381
WashU [75]	USA	32	31	63	23	40	63	20	37	57	20	27	47	20	27	47
Total		1628	3895	5523	1357	4319	5688	1236	3715	4956	1020	2999	4019	1112	3282	4394

See Supplementary Information for an explanation of differences between the columns for “pre-registration” and “initial number of images”.

*GAD* generalized anxiety disorder, *HC* healthy controls, *ABCD* Adolescent Brain Cognitive Development Study, *BHRCS* Brazilian High Risk Cohort Study, *CMI-HBN* Child Mind Institute Healthy Brain Network, *IOL* Institute of Living, *PROTAIA* Anxiety Disorders Program for Child and Adolescent Psychiatry, *SDAN* Section on Development and Affective Neuroscience, *SHIP* Study of Health in Pomerania, *SNFA* Section on Neurobiology of Fear and Anxiety, *UCSD* University of California – San Diego, *UPenn* University of Pennsylvania, *WashU* Washington University.

^a^For 12 participants in the initial number of images there were no behavioral data available, 5 of these participants had images of high quality.

**Table 2:** Reasons for exclusion of participants.

Site	Initial number of images	QC (visual or auto)	Freesurfer failed	Subfield analysis failed	Not in covariates file	“Patients” without GAD^a^	“HC” with other disorder^b^	Comorbid psychosis	Missing age	Missing MDD, OCD, PTSD and SUD	Missing IQ and/or Edu	Participants in main analysis	Participants in secondary analysis
ABCD	1600	140	2	2	0	0	0	0	5	0	0	1451	1451
Barcelona	91	0	0	0	0	0	1	0	0	0	1	89	89
Baylor	228	0	0	0	0	0	0	0	0	0	0	228	228
BHRCS	781^c^	238	1	0	5	58	84	0	0	0	7	388	388
Boystown	95	1	0	0	0	0	1	0	0	0	0	93	93
Chicago-Milad	43	0	0	0	0	0	0	0	0	0	0	43	43
Chicago-Phan	147	3	0	0	0	24	0	0	0	0	0	120	120
Cincinnati	21	16	0	0	0	0	2	0	0	0	0	–	3
CMI-HBN	290	48	11	0	0	0	0	0	0	0	138	93	93
Dresden	94	0	0	0	0	0	0	0	0	0	0	94	94
Duke	45	20	0	0	0	2	0	0	0	0	0	23	23
Harvard	260	38	0	0	0	57	0	0	0	0	2	163	163
Houston	273	63	3	0	0	0	0	0	0	0	17	–	190
IOL	64	16	0	0	0	7	0	0	0	0	1	40	40
Milan	98	1	0	0	0	0	2	0	0	0	28	67	67
Muenster	54	0	0	0	0	0	0	0	0	0	1	53	53
Pittsburgh- Andreescu	79	16	0	0	0	0	0	0	0	0	4	59	59
Pittsburgh-Price	69	0	0	0	0	11	0	0	0	0	3	–	55
PROTAIA	44	6	0	0	0	5	9	1	0	0	9	–	14
SanRaffaele	92	0	1	0	0	0	0	0	0	0	0	–	91
SDAN	406	28	0	0	0	0	0	0	0	0	11	367	367
SHIP	36	0	0	0	0	0	0	0	0	2	0	36	34
SNFA	63	25	0	0	0	2	0	0	0	0	12	–	24
StonyBrook	61	0	1	0	0	0	0	0	0	0	0	60	60
Sussex	40	0	0	0	0	0	0	0	0	0	0	40	40
UCSD	96	6	0	0	0	0	0	0	0	0	6	84	84
UPenn	455	61	0	0	0	0	0	13	0	0	0	381	381
WashU	63	6	0	0	0	7	3	0	0	0	0	47	47
Total	5688	732	19	2	5	173	100	14	5	2	240	4019	4394

*QC* quality checking, *auto* automated, *GAD* generalized anxiety disorder, *HC* healthy controls, *MDD* major depressive disorder, *OCD* obsessive-compulsive disorder, *PTSD* post-traumatic stress disorder, *SUD* substance use dependence, *Edu* education in years, *ABCD* Adolescent Brain Cognitive Development Study, *BHRCS* Brazilian High Risk Cohort Study, *CMI-HBN* Child Mind Institute Healthy Brain Network, *IOL* Institute of Living, *PROTAIA* Anxiety Disorders Program for Child and Adolescent Psychiatry, *SDAN* Section on Development and Affective Neuroscience, *SHIP* Study of Health in Pomerania, *SNFA* Section on Neurobiology of Fear and Anxiety, *UCSD* University of California – San Diego, *UPenn* University of Pennsylvania, *WashU* Washington University.

^a^These participants were classified as patients in the “number of images of high quality” in Table 1, but did not have a GAD diagnosis.

^b^For 12 participants in the initial number of images there were no behavioral data available, 5 of these participants had images of high quality.

^c^These participants were classified as HC in the “number of images of high quality” in Table 1, but had a diagnosis other than GAD.

**Table 3:** Descriptive statistics for sex, age, IQ, and years of education for all sites that were included in the main analysis.

Site	Total		Healthy controls									Individuals with GAD											
	*n*	*n*	% F	Min age	Max age	Mean age	SD age	Mean IQ	SD IQ	Mean Edu	SD Edu	Current GAD	% F	LT GAD	% F	Min age	Max age	Mean age	SD age	Mean IQ	SD IQ	Mean Edu	SD Edu
ABCD	1451	1347	53.8	8.9	11.0	10.00	0.62			4.37	0.76	20	60.0	84	56.0	9.0	10.9	10.12	0.66			4.34	0.80
Barcelona	89	58	69.0	18.0	40.0	22.07	4.37			14.45	2.14	30	66.7	1	100	18.0	33.0	23.13	4.57			14.61	2.22
Baylor	228	130	50.8	12.0	79.0	24.26	12.84					98	41.8	0	–	18.0	56.0	28.85^a^	9.23				
BHRCS	388	373	45.6	5.0	14.0	9.66	1.84	102.58	16.52	4.19	1.69	15	66.7	0	–	7.0	11.0	9.87	1.46	102.60	23.00	4.00	1.25
Boystown	93	44	52.3	13.0	18.0	15.39	1.62	105.23	10.23			49	59.2	0	–	13.0	18.0	15.80	1.37	102.53	13.47		
Chicago-Milad	43	16	25.0	18.0	59.0	32.38	13.39			16.44	2.58	27	66.7	0	–	19.0	51.0	30.26	9.90			16.52	2.31
Chicago-Phan	120	42	54.8	18.0	60.0	25.21	9.77			15.79	3.06	78	73.1	0	–	18.0	58.0	26.90	8.69			16.26	3.13
CMI-HBN	93	54	51.9	6.2	19.3	10.18	2.85	107.00	15.88	6.11	2.84	39	64.1	0	–	6.9	21.7	13.27^a^	3.61	104.62	15.62	9.10^a^	3.51
Dresden	94	47	68.1	19.0	50.0	28.89	8.40			12.13	0.90	47	76.6	0	–	18.0	51.0	30.23	10.05			12.04	1.04
Duke	23	12	58.3	6.0	10.0	7.83	1.03	104.00	12.86			6	66.7	5	100	6.0	8.0	6.73^a^	0.65	105.45	13.00		
Harvard	163	41	63.4	18.0	33.0	25.46	3.92	114.15	7.91	16.59	1.95	122	73.0	0	–	18.0	36.0	24.89	4.25	112.88	11.82	16.07	2.34
IOL	40	16	81.3	22.0	63.0	39.25	15.04	103.44	8.10			24	75.0	0	–	21.0	64.0	41.29	13.34	101.21	7.48		
Milan	67	38	52.6	21.0	64.0	36.05	12.85	125.39	5.69	12.28	4.42	29	58.6	0	–	20.9	69.9	43.18^a^	14.54	120.21^a^	10.41	10.80	4.24
Muenster	53	29	58.6	19.0	55.0	27.83	9.20			12.90	0.49	24	75.0	0	–	20.0	56.0	29.21	10.34			12.46	1.74
Pittsburgh- Andreescu	59	32	56.3	19.0	90.0	57.06	18.04			15.88	3.79	27	77.8	0	–	23.0	79.0	54.04	18.22			15.56	2.49
SDAN	367	219	57.1	8.4	47.2	17.07	6.43	114.09	11.85			129	64.3	19	73.7	8.1	50.3	16.11	8.61	113.53	13.70		
SHIP	36	24	75.0	41.0	70.0	57.17	8.25			10.13	1.55	0	–	12	75.0	41.0	67.0	56.25	7.82			10.13	1.58
StonyBrook	60	20	100	19.0	44.0	21.50	5.75					40	100	0	–	18.0	49.0	22.98	6.04				
Sussex	40	21	85.7	19.0	55.0	28.67	9.45			12.14	2.57	19	89.5	0	–	18.0	44.0	29.79	7.00			12.89	1.82
UCSD	84	39	66.7	18.0	52.0	23.67	8.17			13.92	2.06	45	75.6	0	–	18.0	53.0	29.09^a^	11.22			14.51	2.29
UPenn	381	370	50.0	8.0	23.0	14.84	3.93	105.73	16.19	8.19	3.79	0	–	11	63.6	8.0	19.0	16.36	3.32	107.45	18.09	9.91	3.36
WashU	47	27	48.1	8.0	12.0	9.85	1.06					20	65.0	0	–	8.0	12.0	10.00	1.56				
Total	4019	2999	53.9	5.0	90.0	14.76	10.02	107.22	15.70	6.40	4.08	888	67.8	132	62.9	6.0	79.0	23.65^a^	13.15	110.43^a^	14.33	12.26	5.01

*GAD* generalized anxiety disorder, *F* female, *Min* minimum, *Max* maximum, *SD* standard deviation, *IQ* intelligence quotient, *Edu* education in years, *LT* lifetime, *ABCD* Adolescent Brain Cognitive Development Study, *BHRCS* Brazilian High Risk Cohort Study, *CMI-HBN* Child Mind Institute Healthy Brain Network, *IOL* Institute of Living, *SDAN* Section on Development and Affective Neuroscience, *SHIP* Study of Health in Pomerania, *UCSD* University of California – San Diego, *UPenn* University of Pennsylvania, *WashU* Washington University.

^a^Indicates a significant difference between individuals with GAD and healthy controls.

**Table 4:** Other diagnoses in individuals with GAD for all sites that were included in the main analysis.

Site	SAD		PD		AG		SPH		Other ANXD		MDD		OCD		PTSD		SUD		OtherD	
	Cur	LT	Cur	LT	Cur	LT	Cur	LT	Cur	LT	Cur	LT	Cur	LT	Cur	LT	Cur	LT	Cur	LT
ABCD	4	16	0	3	0	1	21	30	4	45	3	10	18	5	0	8	1	0	61	28
Barcelona	3	0	0	0	0	0	1	0	0	0	0	2	0	0	0	0	0	0	1	1
Baylor	26	0	3	2	2	1	1	1	0	0	62	1	5	3	14	2	50	7	80	1
BHRCS	1	0	0	0	0	0	2	0	1	4	4	0	0	0	0	0	NA	NA	5	0
Boystown	30	0	NA	NA	NA	NA	NA	NA	0	0	19	0	NA	NA	16	0	NA	NA	0	0
Chicago-Milad	5	0	5	0	2	0	6	0	NA	NA	NA	NA	NA	NA	NA	NA	NA	NA	NA	NA
Chicago-Phan	50	0	21	0	6	0	14	0	0	0	42	1	2	0	14	2	1	0	3	0
CMI-HBN	9	0	0	0	0	0	6	0	5	0	14	0	0	0	0	0	3	0	NA	NA
Dresden	6	0	7	3	7	1	11	0	2	1	12	6	0	0	0	2	0	0	13	5
Duke	1	1	NA	NA	NA	NA	NA	NA	2	1	NA	NA	NA	NA	NA	NA	NA	NA	NA	NA
Harvard	75	2	17	12	14	3	25	2	*NA*	*NA*	29	64	14	4	10	6	0	5	13	8
IOL	6	*NA*	2	*NA*	1	*NA*	0	*NA*	0	*NA*	9	*NA*	0	*NA*	0	*NA*	0	*NA*	2	*NA*
Milan	0	0	0	5	0	0	0	0	2	1	0	8	0	0	0	0	0	1	0	2
Muenster	NA	NA	NA	NA	NA	NA	NA	NA	NA	NA	NA	NA	NA	NA	NA	NA	NA	NA	NA	NA
Pittsburgh- Andreescu	2	0	4	1	0	0	0	0	*NA*	*NA*	0	5	0	0	1	0	1	0	2	4
SHIP	0 (1 NA*)*	3 *(*1 NA*)*	0	2	0	1	0	4	0	0	0 (2 NA*)*	8 (2 NA*)*	0 (2 NA*)*	0 (2 *NA)*	0	0	0 (1 *NA)*	4 (1 *NA)*	0	0
SDAN	80	12	2	0	0	0	46	0	0 (*23* NA*)*	0 (*23* NA*)*	2	2	0	0	0	0	0	0	6 (23 NA*)*	1 (23 NA*)*
StonyBrook	0	0	0	0	0	0	0	0	0	0	20	0	0	0	0	0	0	0	0	0
Sussex	0	0	0	0	0	0	0	0	0	0	0	0	0	0	0	0	0	0	0	0
UCSD	20	4	4	2	4	0	NA	NA	0	0	8	8	2	0	0 (14 NA*)*	2 (14 NA)	0	3	0	0
UPenn	0	4	0	1	0	1	0	5	0	4	0	7	0	2	0	4	NA	NA	0	8
WashU	9	0	0	0	0	0	0	1	3	1	3	0	0	0	0	0	0	0	3	6
Total	327	42	65	31	36	8	133	43	19	57	227	122	41	14	55	26	56	20	189	64

*SAD* social anxiety disorder, *PD* panic disorder, *AG* agoraphobia, *SPH* specific phobia, *Other ANXD* other anxiety disorder, *MDD* major depressive disorder, *OCD* obsessive-compulsive disorder, *PTSD* post-traumatic stress disorder, *SUD* substance use dependence, *OtherD* other disorder, *Cur* current, *LT* lifetime, *NA* not available, *ABCD* Adolescent Brain Cognitive Development Study, *BHRCS* Brazilian High Risk Cohort Study, *CMI-HBN* Child Mind Institute Healthy Brain Network, *IOL* Institute of Living, *SDAN* Section on Development and Affective Neuroscience, *SHIP* Study of Health in Pomerania, *UCSD* University of California – San Diego, *UPenn* University of Pennsylvania, *WashU* Washington University.

**Table 5:** Descriptive statistics for medication at scan for all sites that were included in the main analysis.

Site	Total		Healthy controls				Individuals with GAD					
	*n*	*n*	SSRI/SNRI	Benzo	Apsy	Other Med	Current GAD	LT GAD	SSRI/SNRI	Benzo	Apsy	Other Med
ABCD	1451	1347	1	0	0	94	20	84	8	1	1	25
Barcelona	89	58	0	0	0	0	30	1	0	1	0	0
Baylor	228	130	0	0	0	0	98	0	53 (23 NA)	32 (23 NA)	20 (23 NA)	66 (23 NA)
BHRCS	388	373	0	NA	NA	3	15	0	0	NA	NA	0
Boystown	93	44	0	0	0	1	49	0	16	1	13	11
Chicago-Milad	43	16	NA	NA	NA	NA	27	0	NA	NA	NA	NA
Chicago-Phan	120	42	0	0	0	0	78	0	0	0	0	0
CMI-HBN	93	54	0 (30 NA)	*NA*	0 (30 *NA*)	4 (30 *NA*)	39	0	2 (25 NA)	NA	0 (25 NA)	3 (25 NA)
Dresden	94	47	0	0	0	0	47	0	0	0	0	1
Duke	23	12	NA	NA	NA	NA	6	5	NA	NA	NA	NA
Harvard	163	41	0	0	0	0	122	0	24	15	0	9
IOL	40	16	0	0	0	0	24	0	8	9	3	1
Milan	67	38	2	0	0	2	29	0	15	7	1	2
Muenster	53	29	0	0	0	0	24	0	8	0	0	2
Pittsburgh-Andreescu	59	32	0	0	0	0	27	0	0	0	0	0
SDAN	367	219	0	0	0	0	129	19	0	0	0	0
SHIP	36	24	1	0	0	5	0	12	4	0	0	2
StonyBrook	60	20	0	0	0	0	40	0	0	0	0	0
Sussex	40	21	0	0	0	0	19	0	3	0	0	1
UCSD	84	39	0	0	0	0	45	0	0	0	0	0
UPenn	381	370	4	1	0	12	0	11	3	1	2	2
WashU	47	27	0	0	0	0	20	0	0	0	0	0
Total	4019	2999	8	1	0	121	888	132	144	67	40	125

*GAD* generalized anxiety disorder, *LT* lifetime, *SSRI* selective serotonin reuptake inhibitor, *SNRI* serotonin and norepinephrine reuptake inhibitors, *Benzo* benzodiazepines, *Apsy* antipsychotics, *Other med* other psychotropic medication, *NA* not available, *ABCD* Adolescent Brain Cognitive Development Study, *BHRCS* Brazilian High Risk Cohort Study, *CMI-HBN* Child Mind Institute Healthy Brain Network, *IOL* Institute of Living, *SDAN* Section on Development and Affective Neuroscience, *SHIP* Study of Health in Pomerania, *UCSD* University of California – San Diego, *UPenn* University of Pennsylvania, *WashU* Washington University.

### Non-imaging data

All research sites were asked to provide information with respect to several variables of possible interest, such as demographic information (age, sex, IQ, education in years), diagnoses, and information from a clinical interview concerning anxiety (GAD, social anxiety disorder [SAD], panic disorder [PD], agoraphobia [AG], specific phobia [SPH], any other anxiety disorder, age of onset of anxiety disorders) and other disorders (MDD, obsessive-compulsive disorder [OCD], post-traumatic stress disorder [PTSD], substance use dependence [SUD], other psychiatric disorders, age of onset of other disorders), psychotropic medication use at the time of scan (selective serotonin reuptake inhibitor [SSRI], serotonin and norepinephrine reuptake inhibitor [SNRI], benzodiazepines, antipsychotic, other medication, and duration of medication currently used), and several questionnaires measuring continuous anxiety symptoms (see Supplementary Information). Insufficient data were available for analyses with continuous anxiety symptoms. Availability of these variables varied per research site. If the information on medication was missing for some participants within a site, a regressor for “Missing Medication” was added (this was the case for Baylor and CMI-HBN). If the information on medication was missing for all participants within a site, medication was not included as an independent variable in the analyses for that site.

### Image processing

All raw structural MRI images that were received were organized according to the Brain Imaging Data Structure (BIDS) specification and MRI Quality Control (MRIQC) [34] was used for quality checking. All images were subsequently processed with FreeSurfer version 6.0.0 [35] to compute regional measures of cortical thickness, cortical surface area, and subcortical volume. For participants with multiple images available, we selected the image with the highest quality based on the Euler number [36], which is calculated separately for left and right hemispheres. To compare a single value across multiple images, we first selected the worst (farthest from zero, lowest quality) Euler number per image. Then, we selected the image with the best (closer to zero, highest quality) Euler number. All data were visually inspected for gross over- or underestimation of the white/pial surfaces (largely due to motion artifacts). We also performed a semi-automated quality checking of the data by using the ratio between the Euler characteristic and the number of vertices in the surfaces before topology correction, defining site-specific thresholds using a ROC curve constructed using the results of the visual inspection [23]. We resampled the cortical measurements of thickness and area to an icosahedron recursively subdivided four times (fsaverage4), which was used as a common grid for interpolation [37]. Table 2 shows the number of participants excluded based on visual and automatic quality checking.

### Statistical analysis

We compared cortical thickness, cortical surface area, and subcortical volume between individuals with GAD and healthy controls, and examined interactions with age and sex. The dependent variables in the main analysis were cortical thickness and surface area of the 68 regions of the Desikan–Killiany parcellation [38], as well as subcortical volumes for 16 subcortical regions [35]. Two sets of independent variables were considered, each in its own model. The first set consisted of GAD, sex, age, age-squared, and their interactions, with covariates comprising IQ, years of education, medication use at the time of the scan, each of the comorbid disorders (SAD, PD, AG, SPH, MDD, OCD, PTSD, SUD), and scanner. The second model was the same as the first, but further included global brain measures (i.e., total surface area, mean cortical thickness, and total intracranial volume) as nuisance variables. Both models in this main analysis used random slopes (per site) and random intercepts (per scanner); see Supplementary Table 4 for an overview of the analysis. Variance groups, one per scanner, were used to accommodate the possibility of different variances across scanners (heteroscedasticity; smallest variance group had two observations). Together with permutation testing, this eschews the need for explicit data harmonization. We tested six contrasts per model: main effect of GAD (positive and negative), two-way interaction between GAD and sex (positive and negative), two-way interaction between GAD and age, and the three-way interaction between GAD, age, and sex. The linear and quadratic effects of age were combined using an *F*-test. All analyses were performed using the software Permutation Analysis of Linear Models (PALM)^3^ with 500 permutations. The *p*-values were computed after fitting a generalized Pareto distribution to the tail of the permutation distribution [39] thus dispensing with the need of performing a computationally prohibitive large number of permutations. We repeated this main analysis with vertex-wise cortical surface area and thickness as dependent variables (2562 vertices). Independent variables, variance groups, contrasts, and the number of permutations remained the same.

We used family-wise error rate (FWER) correction to address multiple testing. Correction considered all tests within each modality (i.e., 68 cortical regions each for cortical thickness and surface area, and 16 subcortical volumes), all three sets of modalities, and all 12 contrasts. As the correction considers all sets of modalities (or dependent variables) and all contrasts, it is termed MC-FWER (family-wise error rate across modalities and contrasts) [40]. Results at lower levels of correction for multiple testing (e.g., only within a modality, or only across contrasts) are reported in the Supplementary Information (Supplementary Figs. 1 and 2).

All sites provided information on GAD, age, and sex, but the inclusion of the other independent variables varied across sites according to data availability (see Supplementary Table 5 for an overview of the exact independent variables included per site). Participants with missing values in the independent variables (exact variables differed per site) were excluded. Ultimately, the main analysis included 1020 individuals with GAD and 2999 healthy controls. Because 192 participants had to be excluded from the main analysis due to missing IQ and/or education in years, we repeated the main analysis with these two variables removed for all sites; the respective results for the regional and vertex-wise data are reported in the Supplementary Information.

In addition, we ran a secondary analysis with the same dependent and independent variables, but this time using fixed slopes across sites, while keeping the random intercepts per scanner. This analysis allowed the inclusion of more sites and participants, but it assumes that effects are the same (fixed) across all sites. This secondary analysis included 1112 individuals with GAD and 3282 healthy controls. Exploratory analyses including the volume of gray matter within subcortical structures and the gray-white matter contrast are reported in the Supplementary Information.

## Results

### Main analysis

**Fig. 2:**
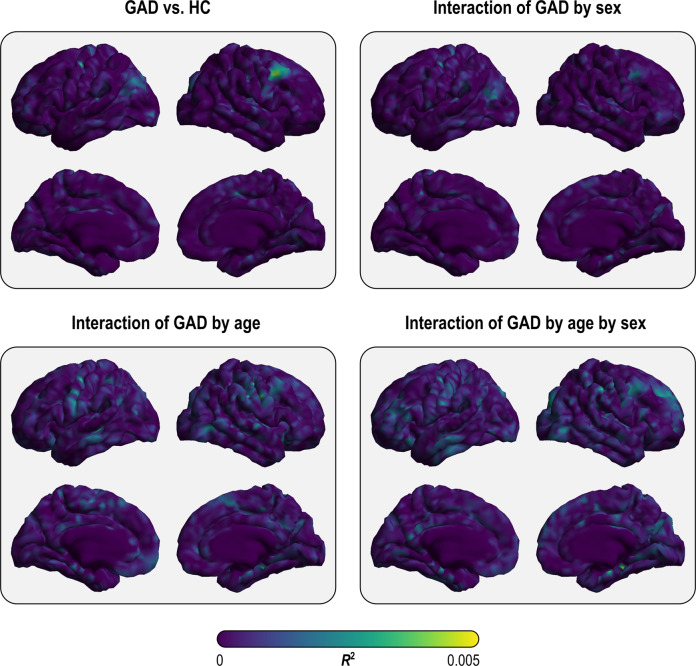
Effect sizes from the main analysis for vertex-wise cortical surface area with the design that included global brain measures as nuisance variables. None of these was statistically significant.

**Fig. 3:**
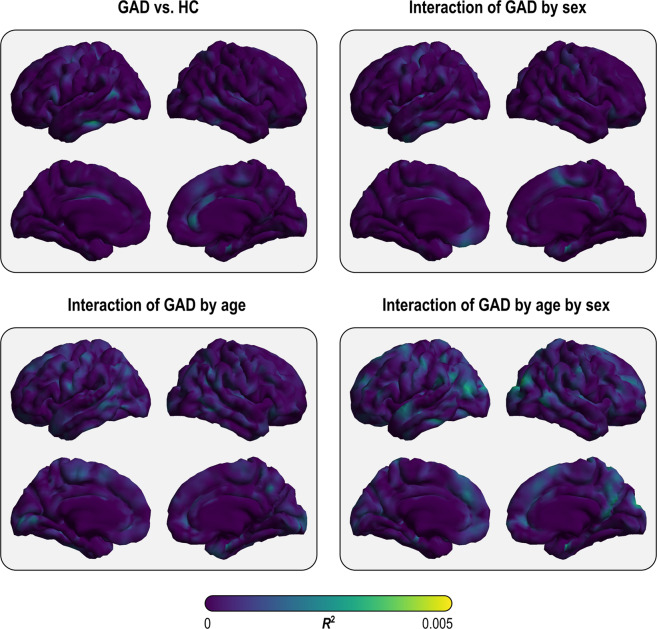
Effect sizes from the main analysis for vertex-wise cortical thickness with the design that included global brain measures as nuisance variables. None of these was statistically significant.

This study compared regional and vertex-wise cortical thickness, cortical surface area, and subcortical volume between individuals with GAD (*n* = 1020) and healthy controls (*n* = 2999) while also examining interactions between GAD, age, and sex. The analysis modeled random slopes for all independent variables per site and random intercepts per scanner. No effects of GAD, nor interactions between GAD, age, or sex on the regional and vertex-wise cortical surface area, cortical thickness, and subcortical volume were significant (see Figs. 2, 3 and Supplementary Figs. 3, 4 for vertex-wise effect sizes). The results remained non-significant when analyses were performed (a) with only the basic independent variables (GAD, age, sex, and their interactions) and (b) when adding the interaction between GAD and medication. The results for the main effects of medication and comorbid disorders and the interaction between GAD and medication were also non-significant.

### Secondary analysis

**Fig. 4:**
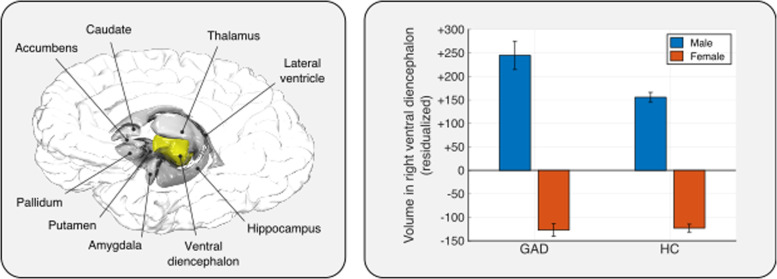
An interaction between generalized anxiety disorder (GAD) and sex in volume in the right ventral diencephalon was observed; male individuals with GAD showed greater volume (mm^3^) compared to male healthy controls, whereas there was no difference between the groups for females. Figure shows data after nuisance variables have been considered (residuals). Average volume of the right ventral diencephalon across individuals with GAD and HC: 3988.6 mm^3^. Note: Error bars reflect standard error.

The secondary analysis included more participants (1112 individuals with GAD and 3282 healthy controls) and implemented approaches more similar to those in other reports from the ENIGMA collaboration. These analyses included fixed slopes for all independent variables and random intercepts per scanner. For the regional data, a significant negative interaction was found between GAD and sex in the volume of the right ventral diencephalon (*R*^2^ = 0.006, *p*_MC-FWER_ = 0.0496 for the whole model fit), in the model without global brain measures as nuisance variables. Male individuals with GAD showed greater volume in the right ventral diencephalon compared to male healthy controls, whereas there was no difference between the groups for females (Fig. 4). The same secondary analysis with fixed slopes for all independent variables and random intercepts per scanner was performed for vertex-wise cortical surface area and thickness data. There were no significant effects of GAD, nor interactions between GAD, age, or sex.

## Discussion

The current study compared regional and vertex-wise cortical thickness, cortical surface area, and subcortical volume between individuals with GAD and healthy controls. Data from 28 sites were combined in a pre-registered analysis that used random slopes and random intercepts to model cross-site heterogeneity. The main analysis showed no effect of GAD on indices of brain structure, nor interactions among GAD, age, or sex. We also conducted a secondary analysis with fixed slopes and random intercepts. This secondary analysis included more sites and thus more participants. This secondary analysis indicated that males with GAD have greater volume, on average, in the right ventral diencephalon compared to healthy males, whereas female individuals with GAD and healthy females did not differ.

Regional and vertex-wise indices of brain structure did not differ between individuals with GAD and healthy controls in the main analysis after multiple comparison corrections. When we did not fully correct for multiple testing, by ignoring the multiplicity of contrasts, there was an interaction between GAD and sex in the left lateral orbitofrontal cortex surface area (Supplementary Results). Prior studies have shown mixed results on the effect of GAD on cortical thickness and surface area [18–20], whereas some studies using VBM have revealed altered gray matter volume in the PFC, amygdala, and hippocampus [15–17]. Small sample sizes and analytical and clinical heterogeneity may account for differences across studies. Here, we leveraged a mega-analysis to mitigate these challenges and found no effect of GAD when accounting for comorbid disorders. The null finding in this study might indicate that these indices of brain structure do not differentiate individuals with GAD from healthy controls. In contrast, ENIGMA studies on MDD have shown significant differences between individuals with MDD and healthy comparisons in hippocampal volume and cortical thickness in bilateral medial OFC, cingulate cortex, insula, and temporal lobes. This could indicate that MDD is more related to structural brain differences than GAD, but this should be confirmed in future studies combining data. Future mega-analyses in GAD could focus on other imaging modalities (e.g., resting-state fMRI, task-based fMRI) or finer imaging phenotypes (e.g., subfields, shape analysis), combine data across imaging and other data types, or use structural covariance analysis or other higher-order constructs for better group differentiation. Some of these analyses have already been started within the ENIGMA-Anxiety Working Group [6, 41].

The secondary analysis with fixed slopes and random intercepts indicated that male individuals with GAD had, on average, greater volume in the right ventral diencephalon compared to male healthy controls, whereas there was no difference between groups for females. The effect size was relatively small, which may explain why this effect arose only in the secondary analysis with more participants and fewer variables in the model. The ventral diencephalon includes the hypothalamus^4^, which plays an important role in the neuroendocrine stress response [42]. Previous studies of GAD found lower hypothalamic volumes [43], and these volumes were negatively associated with anxiety severity in healthy adults [44]. However, these findings focus on the hypothalamus specifically rather than the broader ventral diencephalon region [43], and these samples included mostly females with GAD. Our finding could represent an example of the “gender paradox” hypothesis. This hypothesis posits that across psychiatric disorders, the less frequently affected sex is the one that manifests more severe features of the disorder [45]. This finding is also in line with studies showing differences in structural connectivity in boys but not girls with anxiety disorders [46].

The age range in this sample was large. Some of the largest sites (ABCD, BHRCS) contributed mainly data from young healthy controls, even though the median onset of GAD is in adulthood. We accounted for this by including age and interactions with age in the analysis. Additionally, quadratic effects of age were added because age effects might not be linear [47]. However, nonlinearities that were not modeled could have influenced the data. There might be differences between childhood-onset and adult-onset GAD, but not enough data on the age of onset was available to investigate this further. Hence, interpretation should be in the light of the composition of the sample, which is not a random draw from any specific population, and the data available for analysis. Future mega-analyses could try to collect more detailed clinical data to further investigate childhood-onset and adult-onset GAD.

A few limitations should be noted. First, individuals with current and lifetime GAD were grouped together in the analysis, which could have increased heterogeneity in the GAD group. In addition, the distinction between individuals between current and lifetime GAD might be particularly difficult in the 9–10-year-old children from the ABCD data set. Only 12.9% of the individuals had a diagnosis of lifetime (and not current) GAD at the time of the scan and the results of the main analysis did not change when only individuals with current GAD were included. Second, methods for collecting imaging and non-imaging data differed across research sites. Even though we have accounted for this in the analysis by including random intercepts and slopes per site, it is possible that residual site-specific non-linear effects may still have been present in the data. Third, the results of the secondary analysis with fixed slopes and random intercepts are mostly influenced by the larger samples, such as the ABCD data set (*n* = 1451). However, the main analysis with random slopes and random intercepts is robust to this type of bias. Fourth, variance groups were not taken into account when estimating effect sizes, so the effect sizes could be diminished. Fifth, data quality might be different between sites, which could influence the results, despite the fact that we took certain aspects of heterogeneity into account in the analyses.

To summarize, there was no effect of GAD on regional or vertex-wise cortical thickness, cortical surface area, and subcortical volume, nor interactions among GAD, age, or sex. This is in line with inconsistent findings from prior studies and the clinical heterogeneity of GAD. The secondary analysis showed an interaction between GAD and sex in the ventral diencephalon. Male individuals with GAD showed greater volume in the right ventral diencephalon compared to male healthy controls, whereas there was no detectable difference between female individuals with GAD and healthy controls. Together, these findings show that associations between indices of brain structure and GAD are small, underscoring the subtlety of its effects and perhaps also the clinical heterogeneity of GAD as a phenotype. Showing these null results in a large mega-analysis is important to inform future studies on GAD to focus on other neuroimaging modalities and/or other phenotyping approaches that favor dimensionality.

### Footnotes

1. A meta-analysis involves the computation of a statistic from several cohorts, prior to merging the statistics into an overall estimate of effect size for a variable of interest. A mega-analysis involves a centralized analysis of individual-level data across a range of cohorts, modeling the effect of each cohort and using all the available data to estimate an overall effect size.
2. We repeated the analyses with only individuals with current GAD (*n* = 881; one participant from the ABCD data set had to be excluded, because they were the only participant from one scanner, resulting in a variance group of 1 observation). Similar to the results from individuals with both current and lifetime GAD, the main analysis showed no significant effects of GAD, nor significant interactions between GAD, age, or sex on the regional and vertex-wise cortical surface area, cortical thickness, and subcortical volume. In addition, the secondary analysis with 982 individuals with current GAD also revealed an interaction between GAD and sex in the volume of the right ventral diencephalon, *R*^2^ = 0.007, *p*_MC-FWER_ = 0.038 (for the whole model fit). However, in contrast to the analysis with individuals with both current and lifetime GAD, the vertex-wise secondary analysis revealed an interaction between GAD and age in cortical surface area in one vertex in the superior frontal gyrus in the model with global brain measures (−21.23, 30.13, 48.52; coordinates from FreeSurfer’s FreeView).
3. https://fsl.fmrib.ox.ac.uk/fsl/fslwiki/PALM.
4. Other ventral diencephalon structures include the mammillary bodies, subthalamic nuclei, *substantia nigra*, red nucleus, lateral and medial geniculate nuclei. Some white matter structures such as the *zona incerta*, *crus cerebri*, lenticular fasciculus, and the medial lemniscus are also included in this region, as well as segments of the optic tract.

## Supplementary information

Supplemental material

Supplemental Tables

## Data Availability

Code for data cleaning and analysis will be made available upon request.

## References

[1] Hibar DP, Westlye LT, Doan NT, Jahanshad N, Cheung JW, Ching C (2018). Cortical abnormalities in bipolar disorder: an MRI analysis of 6503 individuals from the ENIGMA Bipolar Disorder Working Group. Mol Psychiatry.

[2] Schmaal L, Hibar DP, Sämann PG, Hall GB, Baune BT, Jahanshad N (2017). Cortical abnormalities in adults and adolescents with major depression based on brain scans from 20 cohorts worldwide in the ENIGMA Major Depressive Disorder Working Group. Mol Psychiatry.

[3] Boedhoe PSW, Schmaal L, Abe Y, Alonso P, Ameis SH, Anticevic A (2018). Cortical abnormalities associated with pediatric and adult obsessive-compulsive disorder: findings from the ENIGMA Obsessive-Compulsive Disorder Working Group. Am J Psychiatry.

[4] van Erp TG, Hibar DP, Rasmussen JM, Glahn DC, Pearlson GD, Andreassen OA (2016). Subcortical brain volume abnormalities in 2028 individuals with schizophrenia and 2540 healthy controls via the ENIGMA consortium. Mol Psychiatry.

[5] Thompson PM, et al. ENIGMA and global neuroscience: a decade of large-scale studies of the brain in health and disease across more than 40 countries. Transl Psychiatry. 2020;10:100. 10.1038/s41398-020-0705-1 PMC7083923 32198361

[6] Bas‐Hoogendam JM, Groenewold NA, Aghajani M, Freitag GF, Harrewijn A, Hilbert K, et al. ENIGMA-anxiety working group: rationale for and organization of large-scale neuroimaging studies of anxiety disorders. Human Brain Map. 2020;1–30. 10.1002/hbm.25100 PMC8805695 32618421

[7] Wittchen HU (2002). Generalized anxiety disorder: prevalence, burden, and cost to society. Depression Anxiety.

[8] Beesdo-Baum K. Phenomenology of generalized anxiety disorder. In: Simon NM, Hollander E, Rothbaum BO, Stein DJ, editors. Anxiety, trauma and OCD-related disorders. 3rd ed. Washington DC: American Psychiatric Association; 2020. p. 161–75.

[9] Kessler RC, Berglund P, Demler O, Jin R, Walters EE (2005). Lifetime prevalence and age-of-onset distributions’ of DSM-IV disorders in the national comorbidity survey replication. Arch Gen Psychiatry.

[10] Grant BF, Hasin DS, Stinson FS, Dawson DA, June Ruan W, Goldstein RB (2005). Prevalence, correlates, co-morbidity, and comparative disability of DSM-IV generalized anxiety disorder in the USA: results from the National Epiderniologic Survey on Alcohol and Related Conditions. Psychol Med.

[11] Beesdo K, Pine DS, Lieb R, Wittchen HU (2010). Incidence and risk patterns of anxiety and depressive disorders and categorization of generalized anxiety disorder. Arch Gen Psychiatry.

[12] Cerda M, Sagdeo A, Johnson J, Galea S (2010). Genetic and environmental influences on psychiatric comorbidity: a systematic review. J Affect Disord.

[13] Kendler KS (1996). Major depression and generalised anxiety disorder—same genes, (partly) different environments—revisited. Br J Psychiatry.

[14] Kendler KS, Neale MC, Kessler RC, Heath AC, Eaves LJ (1992). Major depression and generalized anxiety disorder—same genes, (partly) different environments. Arch Gen Psychiatry.

[15] Hilbert K, Lueken U, Beesdo-Baum K (2014). Neural structures, functioning and connectivity in generalized anxiety disorder and interaction with neuroendocrine systems: a systematic review. J Affect Disord.

[16] Gold AL, Brotman MA, Adleman NE, Lever SN, Steuber ER, Fromm SJ (2016). Comparing brain morphometry across multiple childhood psychiatric disorders. J Am Acad Child Adolesc Psychiatry.

[17] Kolesar TA, Bilevicius E, Wilson AD, Kornelsen J (2019). Systematic review and meta-analyses of neural structural and functional differences in generalized anxiety disorder and healthy controls using magnetic resonance imaging. NeuroImage.

[18] Molent C, Maggioni E, Cecchetto F, Garzitto M, Piccin S, Bonivento C (2018). Reduced cortical thickness and increased gyrification in generalized anxiety disorder: a 3 T MRI study. Psychol Med.

[19] Andreescu C, Tudorascu D, Sheu LK, Rangarajan A, Butters MA, Walker S (2017). Brain structural changes in late-life generalized anxiety disorder. Psychiatry Res.

[20] Strawn JR, John Wegman C, Dominick KC, Swartz MS, Wehry AM, Patino LR (2014). Cortical surface anatomy in pediatric patients with generalized anxiety disorder. J Anxiety Disord.

[21] Thompson PM, Stein JL, Medland SE, Hibar DP, Vasquez AA, Renteria ME (2014). The ENIGMA Consortium: large-scale collaborative analyses of neuroimaging and genetic data. Brain Imaging Behav.

[22] Boedhoe PSW, Heymans MW, Schmaal L, Abe Y, Alonso P, Ameis SH (2019). An empirical comparison of meta- and mega-analysis with data from the ENIGMA Obsessive-Compulsive Disorder Working Group. Front Neuroinformatics.

[23] Zugman A, et al. Mega-analysis methods in ENIGMA: The experience of the generalized anxiety disorder working group. Human Brain Map. 2020;1–23. 10.1002/hbm.25096 PMC8675407 32596977

[24] Harrewijn A, et al. Comparing cortical and subcortical brain structure between patients with generalized anxiety disorder and healthy comparison subjects—findings from the ENIGMA Generalized Anxiety Disorder Working Group. OSF Preregistration; 2019; 10.17605/OSF.IO/YXAJS.

[25] Canu E, Kostić M, Agosta F, Munjiza A, Ferraro PM, Pesic D (2015). Brain structural abnormalities in patients with major depression with or without generalized anxiety disorder comorbidity. J Neurol.

[26] Cha J, Greenberg T, Song I, Blair Simpson H, Posner J, Mujica-Parodi LR (2016). Abnormal hippocampal structure and function in clinical anxiety and comorbid depression. Hippocampus.

[27] Hilbert K, Pine DS, Muehlhan M, Lueken U, Steudte-Schmiedgen S, Beesdo-Baum K (2015). Gray and white matter volume abnormalities in generalized anxiety disorder by categorical and dimensional characterization. Psychiatry Res.

[28] Cardinale EM, et al. Parsing neurodevelopmental features of irritability and anxiety: Replication and validation of a latent variable approach. Dev Psychopathol. 2019;31:917–29. 10.1017/S095457941900035X PMC7439289 31064595

[29] Gold AL, Steuber ER, White LK, Pacheco J, Sachs JF, Pagliaccio D (2017). Cortical thickness and subcortical gray matter volume in pediatric anxiety disorders. Neuropsychopharmacology.

[30] Casey BJ, Cannonier T, Conley MI, Cohen AO, Barch DM, Heitzeg MM (2018). The Adolescent Brain Cognitive Development (ABCD) study: imaging acquisition across 21 sites. Dev Cogn Neurosci.

[31] Volkow ND, Koob GF, Croyle RT, Bianchi DW, Gordon JA, Koroshetz WJ (2018). The conception of the ABCD study: from substance use to a broad NIH collaboration. Dev Cogn Neurosci.

[32] Alexander LM, et al. An open resource for transdiagnostic research in pediatric mental health and learning disorders. Sci Data. 2017;4:170181. 10.1038/sdata.2017.181 PMC5735921 29257126

[33] Carpenter KLH, et al. Preschool anxiety disorders predict different patterns of amygdala-prefrontal connectivity at school-age. PLoS ONE. 2015;10:1–24. 10.1371/journal.pone.0116854 PMC4308069 25625285

[34] Esteban O, et al. MRIQC: advancing the automatic prediction of image quality in MRI from unseen sites. PLoS ONE. 2017;12:e0184661. 10.1371/journal.pone.0184661 PMC5612458 28945803

[35] Fischl B, Salat DH, Busa E, Albert M, Dieterich M, Haselgrove C (2002). Whole brain segmentation: Automated labeling of neuroanatomical structures in the human brain. Neuron.

[36] Rosen AFG, Roalf DR, Ruparel K, Blake J, Seelaus K, Villa LP (2018). Quantitative assessment of structural image quality. Neuroimage.

[37] Winkler AM, Sabuncu MR, Yeo BT, Fischl B, Greve DN, Kochunov P (2012). Measuring and comparing brain cortical surface area and other areal quantities. Neuroimage.

[38] Desikan RS, Ségonne F, Fischl B, Quinn BT, Dickerson BC, Blacker D (2006). An automated labeling system for subdividing the human cerebral cortex on MRI scans into gyral based regions of interest. NeuroImage.

[39] Winkler AM, Ridgway GR, Douaud G, Nichols TE, Smith SM (2016). Faster permutation inference in brain imaging. Neuroimage.

[40] Winkler AM, Webster MA, Brooks JC, Tracey I, Smith SM, Nichols TE (2016). Non-parametric combination and related permutation tests for neuroimaging. Hum Brain Mapp.

[41] Hilbert K, et al. Cortical and subcortical structural alterations in specific phobia: results from the ENIGMA Specific Phobia Working Group. OSF Preregistration; 2020.

[42] de Kloet ER, Joels M, Holsboer F (2005). Stress and the brain: from adaptation to disease. Nat Rev Neurosci.

[43] Terlevic R, Isola M, Ragogna M, Meduri M, Canalaz F, Perini L (2013). Decreased hypothalamus volumes in generalized anxiety disorder but not in panic disorder. J Affect Disord.

[44] Modi S, Thaploo D, Kumar P, Khushu S (2019). Individual differences in trait anxiety are associated with gray matter alterations in hypothalamus: Preliminary neuroanatomical evidence. Psychiatry Res.

[45] Eme RF (1992). Selective female affliction in the developmental disorders of childhood—a literature-review. J Clin Child Psychol.

[46] Tromp DPM, Williams LE, Fox AS, Oler JA, Roseboom PH, Rogers GM (2019). Altered uncinate fasciculus microstructure in childhood anxiety disorders in boys but not girls. Am J Psychiatry.

[47] Mills KL, Goddings AL, Herting MM, Meuwese R, Blakemore SJ, Crone EA (2016). Structural brain development between childhood and adulthood: convergence across four longitudinal samples. Neuroimage.

[48] Porta-Casteràs D, Fullana MA, Tinoco D, Martínez-Zalacaín I, Pujol J, Palao DJ (2020). Prefrontal-amygdala connectivity in trait anxiety and generalized anxiety disorder: testing the boundaries between healthy and pathological worries. J Affect Disord.

[49] Gosnell SN, et al. Hippocampal volume in psychiatric diagnoses: Should psychiatry biomarker research account for comorbidities? Chronic Stress. 2020;4:2470547020906799. 10.1177/2470547020906799 PMC7219869 32440605

[50] Salum GA, Gadelha A, Pan PM, Moriyama TS, Graeff-Martins AS, Tamanaha AC (2015). High risk cohort study for psychiatric disorders in childhood: rationale, design, methods and preliminary results. Int J Methods Psychiatr Res.

[51] Blair KS, et al. Association of different types of childhood maltreatment with emotional responding and response control among youths. JAMA Netw Open. 2019;2:e194604. 10.1001/jamanetworkopen.2019.4604 PMC6632148 31125109

[52] Blair RJR, White SF, Tyler PM, Johnson K, Lukoff J, Thornton LC (2019). Threat responsiveness as a function of cannabis and alcohol use disorder severity. J Child Adolesc Psychopharmacol.

[53] Marin MF, Hammoud MZ, Klumpp H, Simon NM, Milad MR (2020). Multimodal categorical and dimensional approaches to understanding threat conditioning and its extinction in individuals with anxiety disorders. JAMA Psychiatry.

[54] Gorka SM, Young CB, Klumpp H, Kennedy AE, Francis J, Ajilore O (2019). Emotion-based brain mechanisms and predictors for SSRI and CBT treatment of anxiety and depression: a randomized trial. Neuropsychopharmacology.

[55] Klumpp H, Kinney KL, Kennedy AE, Shankman SA, Langenecker SA, Kumar A (2018). Trait attentional control modulates neurofunctional response to threat distractors in anxiety and depression. J Psychiatr Res.

[56] Strawn JR, Bitter SM, Weber WA, Chu WJ, Whitsel RM, Adler C (2012). Neurocircuitry of generalized anxiety disorder in adolescents: a pilot functional neuroimaging and functional connectivity study. Depress Anxiety.

[57] Wu MJ, Wu HE, Mwangi B, Sanches M, Selvaraj S, Zunta-Soares GB (2015). Prediction of pediatric unipolar depression using multiple neuromorphometric measurements: a pattern classification approach. J Psychiatr Res.

[58] Assaf M, et al. Neural functional architecture and modulation during decision making under uncertainty in individuals with generalized anxiety disorder. Brain Behav. 2018;8:e01015. 10.1002/brb3.1015 PMC6085921 29931835

[59] Diefenbach GJ, Bragdon LB, Zertuche L, Hyatt CJ, Hallion LS, Tolin DF (2016). Repetitive transcranial magnetic stimulation for generalised anxiety disorder: a pilot randomised, double-blind, sham-controlled trial. Br J Psychiatry.

[60] Buff C, Brinkmann L, Neumeister P, Feldker K, Heitmann C, Gathmann B (2016). Specifically altered brain responses to threat in generalized anxiety disorder relative to social anxiety disorder and panic disorder. NeuroImage.

[61] Karim H, Tudorascu DL, Aizenstein H, Walker S, Good R, Andreescu C (2016). Emotion reactivity and cerebrovascular burden in late-life GAD: a neuroimaging study. Am J Geriatr Psychiatry.

[62] Price RB, Beltz AM, Woody ML, Cummings L, Gilchrist D, Siegle GJ (2020). Neural connectivity subtypes predict discrete attentional bias profiles among heterogeneous anxiety patients. Clin Psychol Sci.

[63] Price RB, Cummings L, Gilchrist D, Graur S, Banihashemi L, Kuo SS (2018). Towards personalized, brain-based behavioral intervention for transdiagnostic anxiety: transient neural responses to negative images predict outcomes following a targeted computer-based intervention. J Consult Clin Psychol.

[64] Salum GA, Isolan LR, Bosa VL, Tocchetto AG, Teche SP, Schuch I (2011). The multidimensional evaluation and treatment of anxiety in children and adolescents: rationale, design, methods and preliminary findings. Braz J Psychiatry.

[65] Toazza R, Franco AR, Buchweitz A, Molle RD, Rodrigues DM, Reis RS (2016). Amygdala-based intrinsic functional connectivity and anxiety disorders in adolescents and young adults. Psychiatry Res Neuroimaging.

[66] Gold AL, Abend R, Britton JC, Behrens B, Farber M, Ronkin E (2020). Age differences in the neural correlates of anxiety disorders: an fMRI study of response to learned threat. Am J Psychiatry.

[67] Völzke H, Alte D, Schmidt CO, Radke D, Lorbeer R, Friedrich N (2011). Cohort profile: the study of health in pomerania. Int J Epidemiol.

[68] Balderston NL, Vytal KE, O'Connell K, Torrisi S, Letkiewicz A, Ernst M (2017). Anxiety patients show reduced working memory related dlPFC activation during safety and threat. Depress Anxiety.

[69] Makovac E, Watson DR, Meeten F, Garfinkel SN, Cercignani M, Critchley HD (2016). Amygdala functional connectivity as a longitudinal biomarker of symptom changes in generalized anxiety. Soc Cogn Affect Neurosci.

[70] Makovac E, Meeten F, Watson DR, Herman A, Garfinkel SN, D Critchley H (2016). Alterations in amygdala-prefrontal functional connectivity account for excessive worry and autonomic dysregulation in generalized anxiety disorder. Biol Psychiatry.

[71] Ball TM, Ramsawh HJ, Campbell-Sills L, Paulus MP, Stein MB (2013). Prefrontal dysfunction during emotion regulation in generalized anxiety and panic disorders. Psychol Med.

[72] Fonzo GA, Ramsawh HJ, Flagan TM, Sullivan SG, Simmons AN, Paulus MP (2014). Cognitive-behavioral therapy for generalized anxiety disorder is associated with attenuation of limbic activation to threat-related facial emotions. J Affect Disord.

[73] Calkins ME, Merikangas KR, Moore TM, Burstein M, Behr MA, Satterthwaite TD (2015). The Philadelphia Neurodevelopmental Cohort: constructing a deep phenotyping collaborative. J Child Psychol Psychiatry.

[74] Satterthwaite TD, Elliott MA, Ruparel K, Loughead J, Prabhakaran K, Calkins ME (2014). Neuroimaging of the Philadelphia Neurodevelopmental Cohort. Neuroimage.

[75] Perino MT, et al. Attention alterations in pediatric anxiety: evidence from behavior and neuroimaging. Biol Psychiatry. 2021;89:726–34. 10.1016/j.biopsych.2020.07.016 PMC9166685 33012520

