# Supplementary material for: Cortical and subcortical brain structure in generalized anxiety disorder: findings from 28 research sites in the ENIGMA-Anxiety Working Group
Source: Transl Psychiatry. 2021 Oct 1;11:502. doi: 10.1038/s41398-021-01622-1 (PMC8486763; doi:10.1038/s41398-021-01622-1)
Supplement: Supplementary file 1 — Supplemental material [file 41398_2021_1622_MOESM1_ESM.docx]

Supplementary Information

# Supplementary Methods

## Site-specific information on participants

We received data from 5523 participants before pre-registration^1^. However, there were some small changes after pre-registration (‘pre-registration’ columns in Table 1) and before the start of the preprocessing of the data (‘initial number of images’ columns in Table 1). For the Adolescent Brain Cognitive Development Study (ABCD) dataset^2,3^, we included only individuals with information on sex and age, and with available structural imaging data. Nine participants were excluded because they did not have original (‘rec-orig’) images, only non-uniformity corrected (‘rec-norm’) images. For Barcelona^4^, one individual with generalized anxiety disorder (GAD) was by accident scored as a healthy comparison subject in the pre-registration. For Baylor ^5^, data of 130 healthy controls were received after pre-registration. For the Child Mind Institute Healthy Brain Network (CMI-HBN) dataset^6^, one participant only had a FLAIR image. For the Brazilian High Risk Cohort Study (BHRCS) dataset^7^, pre-registered data included individuals with disorders other than GAD, the ‘initial number of images’ included only individuals with GAD. In addition, no non-imaging data was available for 12 participants with imaging data. For the Section on Development and Affective Neuroscience (SDAN) dataset^8,9^, seven participants were excluded because they were the only participants scanned on an old MRI scanner, one participant was excluded because no image was available, and five participants were added after pre-registration. For the Study of Health in Pomerania (SHIP) dataset^10^, all data was received after pre-registration, adding data from 36 participants.

## Non-imaging data: questionnaires

All research sites were asked to provide as much questionnaire data as possible to measure continuous generalized anxiety symptoms (Hamilton Anxiety Rating Scale^11^, Penn State Worry Questionnaire^12^, Generalized Anxiety Disorder 7-Item questionnaire^13^), generic anxiety symptoms (State Trait Anxiety Inventory^14^, Anxiety Sensitivity Index^15^, Beck Anxiety Inventory^16^), social anxiety symptoms (Liebowitz Social Anxiety Scale^17^), panic disorder/agoraphobia symptoms (Panic and Agoraphobia Scale^18^, Agoraphobic Cognition Questionnaire^19^, Panic Disorder Severity Scale^20^), and depressive symptoms (Beck Depression Inventory II^21^, Hamilton Depression Rating Scale^22^). To measure continuous anxiety and depressive symptoms in pediatric samples, respectively the Screen Child Anxiety Related Disorders^23^ and Children’s Depression Inventory^24^ were used. Not all research sites provided data on all these variables, therefore, these data were not included in the analyses.

## Site-specific information on non-imaging data

In the ABCD dataset, the computerized Kiddie Schedule for Affective Disorders and Schizophrenia (KSADS) was used for parents, and a shortened version of the computerized KSADS was used for children^25^. Individuals from the ABCD dataset were included if they were diagnosed with current or past GAD, based on either the parent-reported KSADS or the child-reported KSADS. The healthy controls in the Baylor dataset were not tested for psychiatric diagnoses; we noted all diagnoses as ‘0’ to be able to include this dataset in the analysis. The CMI-HBN dataset included IQ scores from the Wechsler Adult Intelligence Scale (WAIS^26^), Wechsler Intelligence Scale for Children (WISC^27^), and the Wechsler Abbreviated Scale of Intelligence (WASI^28^). The full-scale IQ score (FSIQ) was used when available, the WAIS processing speed index (“WAIS_Abb_PSI”) was used if the FSIQ was not available. Three participants had both WISC and WASI scores, for these participants the WISC was used because it has more subscales. The dataset from Harvard included two measures of IQ: matrix^29^ and Shipley^30,31^. We included only the Shipley IQ measure because it was available for more participants. BHRCS only recorded ‘SSRI/SNRI’ and ‘other medication’ for medication. CMI-HBN did not collect information on use of benzodiazepines. For one participant in the SanRaffaele dataset, information on use of SSRI/SNRI, antipsychotics and other medication was missing. However, this participant was using benzodiazepines, so was included as using medication. Only medication within 24 hours of the scan was used in the analysis for the ABCD dataset.

## Site-specific information on image processing

Three sites had different types of structural images available: for the ABCD dataset we used the ‘orig’ images because not all participants had a ‘norm’ (bias corrected) image, for the CMI-HBN dataset we used the ‘VNav-norm’, ‘HCP’ and ‘simple T1w’ images, selecting always the same type of sequence for a given scanner, such that both scanner and sequence would deliberately be perfectly confounded and thus, explained by the same scanner-specific regressors. For the Washington University (WashU) dataset we used ‘norm’ (bias corrected) images instead of ‘orig’ images based on the average Euler number across participants across hemispheres (*M*=-42.29 for ‘norm’ and *M*=-47.71 for ‘orig’).

## Site-specific information on statistical analysis

Two sites (Duke and Sussex) included not enough participants to model the quadratic effects of age, so only the linear effects of age were included for these two sites. One site (IOL) did not assess lifetime diagnoses and participants with no current diagnosis were coded as 0 (not having current and/or diagnosis), to be able to include these participants in the analysis.

Only for the datasets from University of Pennsylvania (UPenn) and the Study of Health in Pomerania (SHIP), all comorbid disorders (SAD, PD, AG, SPH, MDD, OCD, and PTSD) had to be combined into one independent variable, because the design would otherwise be rank deficient. In addition, six sites were excluded from the main analysis: two of these sites (Cincinnati and Pittsburgh-Price) included only individuals with GAD, so it was not possible to model the effect of GAD within these sites; data from Houston and San Raffaele were excluded because GAD was fully confounded with MDD; the models for the Anxiety Disorders Program for Child and Adolescent Psychiatry (PROTAIA) and the Section on Neurobiology of Fear and Anxiety (SNFA) were rank deficient due to a low number of individuals with GAD, leading the column that codes diagnosis to be fully represented by linear combinations of nuisance variables.

## Exploratory analyses

Two additional measures of brain structure have been reported in previous studies and were included in exploratory analyses^32,33^. These are the volume of gray matter within subcortical structures and the gray-white matter contrast. For the former partial volume effects for gray matter were computed by segmenting the images into gray matter, white matter and cerebrospinal fluid ^32^ and summing the partial volumes of gray matter over all voxels within each of the subcortical regions. We ran an exploratory analysis with subcortical volume and partial gray matter volume as dependent variables, random slopes (per site) and random intercepts (per scanner) for the same two sets of independent variables as in the main analysis. The results are reported in the Supplementary Information.

For the latter, we computed the gray-white matter contrast for each vertex across the cortex^33^. These were used in an exploratory vertex-wise analysis, along with cortical surface area and cortical thickness as dependent variables, random slopes (per site) and random intercepts (per scanner) again for the two sets of independent variables.

# Supplementary Results

## Main analysis at lower levels of correction for multiple testing

The main analysis included random slopes for all independent variables per site and random intercepts per scanner. The MC-FWER correction considers all sets of modalities (cortical thickness, cortical surface area, and subcortical volume) and all contrasts (see Supplementary Table 1). There were no significant effects at this level of correction as described in the main body of the manuscript. If the multiple testing correction was relaxed by ignoring the multiplicity of contrasts, then one finding arose for the regional data: a negative interaction between GAD and sex in surface area in the left lateral orbitofrontal cortex in the model with global brain measures as nuisance variables (Supplementary Table 1, model 2), *R^2^* = 0.0003, *p*_M-FWER_ = 0.03.

Several findings arose when ignoring the multiplicity of contrasts in the vertex-wise main analysis (Supplementary Figure 1). First, individuals with GAD showed increased cortical surface area in the caudal middle frontal gyrus in the model with global brain measures as nuisance variables. Second, individuals with GAD showed decreased cortical thickness in the inferior parietal cortex in the model without global brain measures and in the supramarginal cortex in the model with global brain measures.

More vertex-wise findings were significant when ignoring both the multiplicity of modalities and of contrasts. Individuals with GAD showed increased cortical thickness in the rostral anterior cingulate cortex in the model without global brain measures and decreased cortical surface area in the postcentral cortex in the model with global brain measures. There was a positive interaction between GAD and sex in the supramarginal cortex and a negative interaction between SAD and sex in the banks of the superior temporal sulcus, both in the model with global brain measures. Finally, there was a three-way interaction between GAD, sex, and age in the parahippocampal cortex in the model without global brain measures.


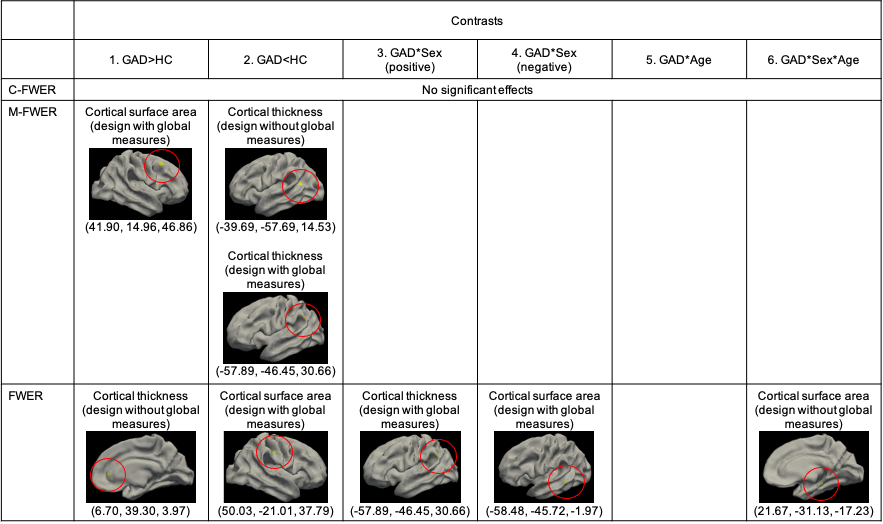


Supplementary Figure 1. Results for the main analysis on vertex-wise data when correcting only across contrasts (C-FWER *p*-values), across modalities (M-FWER *p*-values), or only within one modality (FWER *p*-values). Coordinates are from Freeview (a data visualization tool in FreeSurfer ^34^, on white matter surface).

## Secondary analysis at lower levels of correction for multiple testing

The secondary analysis included fixed slopes for all independent variables and random intercepts per scanner. For the regional findings, a significant negative interaction was found between GAD and sex in the volume of the right ventral diencephalon in the model without global brain measures as nuisance variables, *R^2^* = 0.07, *p*_MC-FWER_ = 0.0496. Other regional findings were seen when ignoring the multiplicity of contrasts. The negative interaction between GAD and sex in the volume of the right ventral diencephalon extended to the model with global brain measures as nuisance variables, *R^2^* = 0.002, *p*_M-FWER_ = 0.02, and to the left ventral diencephalon in the model without global brain measures, *R^2^* = 0.003, *p*_M-FWER_ = 0.045. When ignoring the multiplicity of modalities (but not of contrasts), individuals with GAD showed greater left putamen volume than healthy controls in the model without global brain measures, *R^2^* = 0.002, *p*_C-FWER_ = 0.048. When ignoring both the multiplicity of modalities and of contrasts, the main effect of GAD in the left putamen extended to the model with global brain measures, *R^2^* = 0.001, *p*_FWER_ = 0.01. Moreover, a main effect of GAD was found in the right putamen in models with and without global brain measures, respectively *R^2^* = 0.001, *p*_FWER_ = 0.04 and *R^2^* = 0.002, *p*_FWER_ = 0.01, with individuals with GAD showing greater volume in the right putamen than healthy controls. The negative interaction between GAD and sex, at this lowest level of multiple testing correction, was also significant for the right thalamus, *R^2^* = 0.001, *p*_FWER_ = 0.02, left putamen, *R^2^* = 0.001, *p*_FWER_ = 0.03, left amygdala, *R^2^* = 0.001, *p*_FWER_ = 0.047, and right amygdala, *R^2^* = 0.001, *p*_FWER_ = 0.02, all in the model without global brain measures as nuisance variables. The three-way interaction between GAD, age and sex was significant in the models with and without global brain measures in the right ventral diencephalon, respectively *R^2^* = 0.002, *p*_FWER_ = 0.004 and *R^2^* = 0.002, *p*_FWER_ = 0.03.

Supplementary Figure 2 shows the results for the vertex-wise analyses at lower levels of correction for multiple testing. When ignoring the multiplicity of modalities, there was an interaction between GAD and age in cortical surface area in the pericalcaerine cortex in the model with global brain measures. When ignoring the multiplicity of contrasts, there was a negative interaction between GAD and sex in cortical surface area in the pericalcaerine cortex in the model with global brain measures. There was also a negative interaction between GAD and sex in cortical thickness in the precentral cortex, insula, and medial orbitofrontal cortex (this last vertex was not in the Desikan parcellation, but very close to the medial orbitofrontal cortex) in the model without global brain measures. Finally, there was a three-way interaction between GAD, sex, and age in cortical thickness in the superior frontal cortex (2 verteces) in the model with global brain measures.

More vertex-wise findings were significant when ignoring both the multiplicity of modalities and of contrasts. Individuals with GAD showed increased cortical thickness in the postcentral cortex, precuneus, superior parietal cortex, and inferior parietal cortex in the model without global brain measures. There was also an interaction between GAD and age in cortical thickness in the supramarginal cortex (2 vertices) and a three-way way interaction between GAD, sex, and age in cortical thickness in the precentral cortex and superior frontal cortex (3 vertices), all in the model with global brain measures.


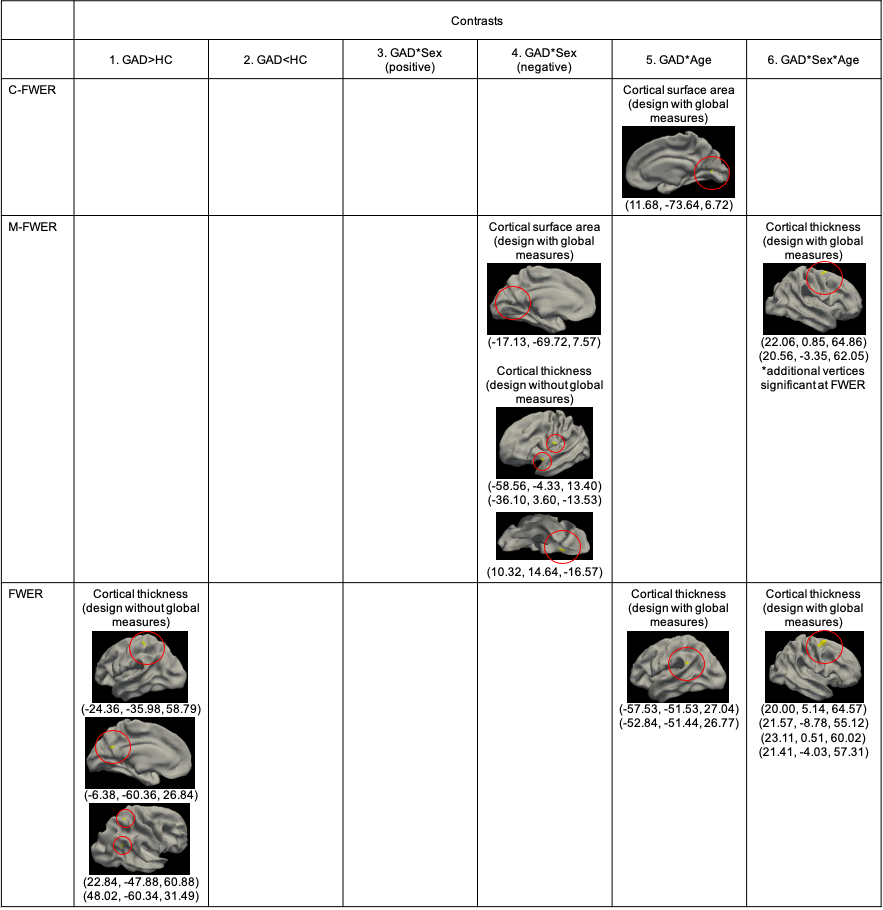


Supplementary Figure 2. Results for the secondary analysis on vertex-wise data when correcting only across contrasts (C-FWER *p*-values), across modalities (M-FWER *p*-values), or only within one modality (FWER *p*-values). Coordinates are from Freeview (a data visualization tool in FreeSurfer^34^, on white matter surface).

## Main analysis without global measures


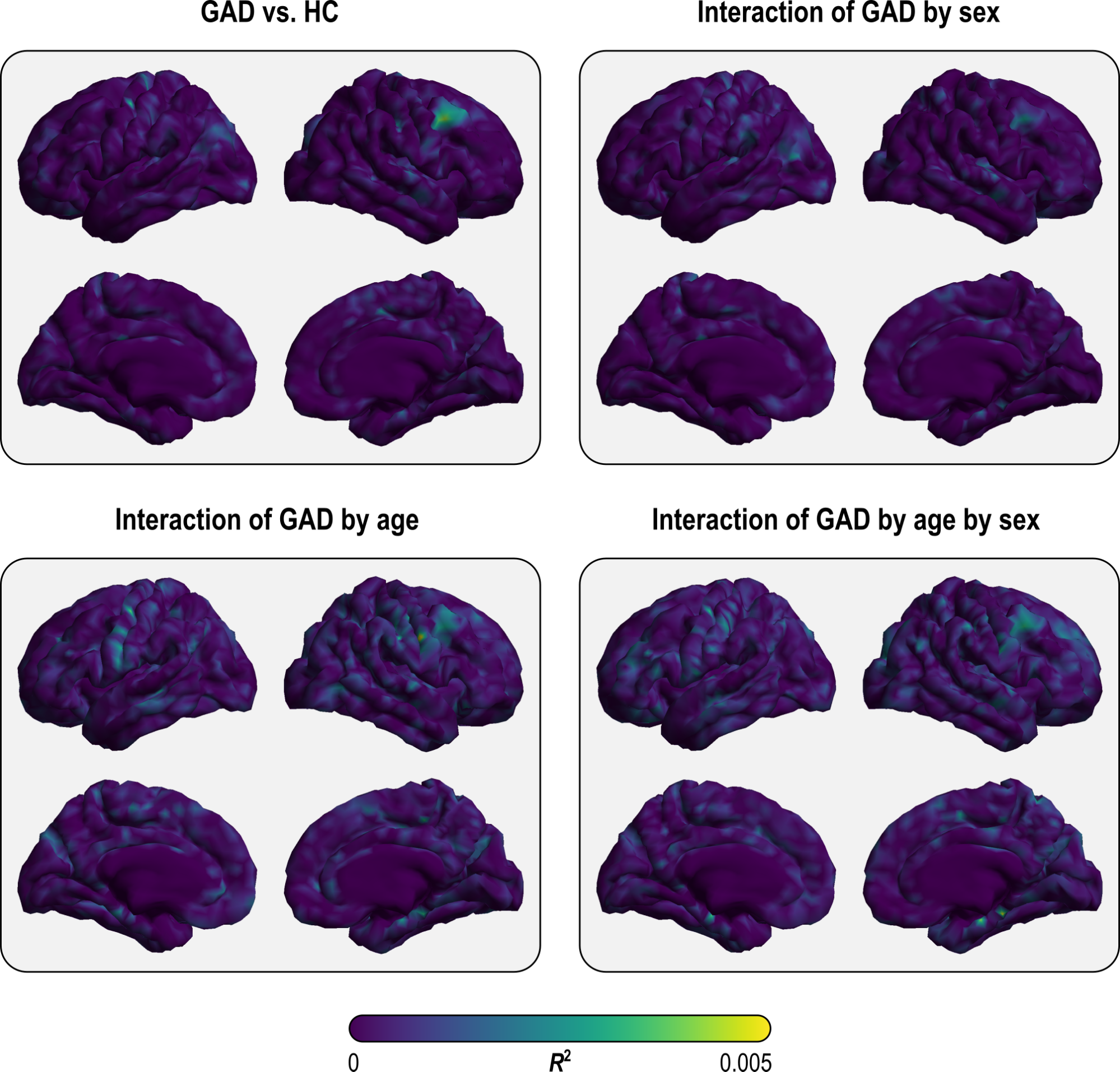
Supplementary Figure 3. Effect sizes from the main analysis for vertex-wise cortical surface area with the design without global brain measures.


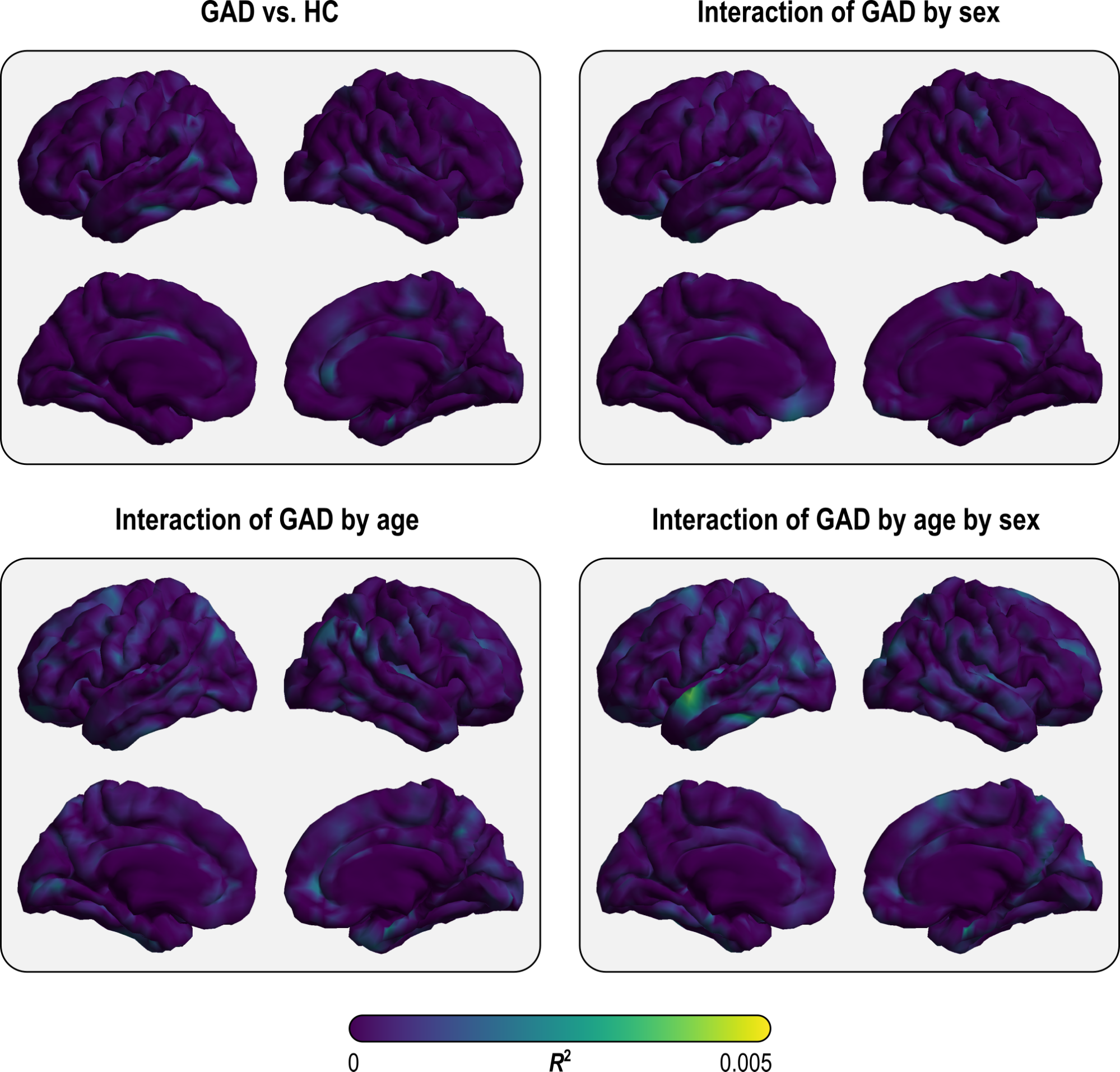
Supplementary Figure 4. Effect sizes from the main analysis for vertex-wise cortical thickness with the design without global brain measures.

## Main analysis without IQ and education

The goal of the current study was to compare cortical thickness, cortical surface area, and subcortical volume between individuals with GAD and healthy controls, and to examine interactions among GAD, age and sex. Random slopes for all independent variables per site and random intercepts per scanner were modeled in this main analysis. 192 participants had to be excluded from the main analysis due to missing IQ and/or education in years, so we repeated the main analysis with these two variables removed from the model for all sites. Similar to the results from the main analysis, no significant effects of GAD, nor interactions between GAD, age or sex were found for cortical thickness, cortical surface area, and subcortical volume in both the regional and vertex-wise analysis.

## Exploratory analysis

We ran an exploratory regional analysis with subcortical volume and partial gray matter volume (both for 16 subcortical regions) as dependent variables, random slopes per site for the two sets of independent variables, and random intercepts per scanner. There were no significant effects of GAD, nor interactions between GAD, age or sex.

In addition, we ran an exploratory vertex-wise analysis with cortical area, thickness and gray-white matter contrast as dependent variables, random slopes per site and random intercepts per scanner for the two sets of independent variables. There were no significant effects of GAD, nor interactions between GAD, age or sex on vertex-wise gray-white matter contrast.

# References

1. Harrewijn A, Cardinale EM, Bas-Hoogendam JM, Groenewold N, Stein DJ, Van der Wee NJA *et al.* Comparing cortical and subcortical brain structure between patients with generalized anxiety disorder and healthy comparison subjects – findings from the ENIGMA Generalized Anxiety Disorder Working Group. *OSF Preregistration* 2019.

2. Casey BJ, Cannonier T, Conley MI, Cohen AO, Barch DM, Heitzeg MM *et al.* The Adolescent Brain Cognitive Development (ABCD) study: Imaging acquisition across 21 sites. *Developmental Cognitive Neuroscience* 2018; **32:** 43-54.

3. Volkow ND, Koob GF, Croyle RT, Bianchi DW, Gordon JA, Koroshetz WJ *et al.* The conception of the ABCD study: From substance use to a broad NIH collaboration. *Developmental Cognitive Neuroscience* 2018; **32:** 4-7.

4. Porta-Casteras D, Fullana MA, Tinoco D, Martinez-Zalacain I, Pujol J, Palao DJ *et al.* Prefrontal-amygdala connectivity in trait anxiety and generalized anxiety disorder: Testing the boundaries between healthy and pathological worries. *Journal of Affective Disorders* 2020; **267:** 211-219.

5. Gosnell SN, Meyer MJ, Jennings C, Ramirez D, Schmidt J, Oldham J *et al.* Hippocampal volume in psychiatric diagnoses: Should psychiatry biomarker research account for comorbidities? *Chronic Stress (Thousand Oaks)* 2020; **4**.

6. Alexander LM, Escalera J, Ai L, Andreotti C, Febre K, Mangone A *et al.* An open resource for transdiagnostic research in pediatric mental health and learning disorders. *Scientific Data* 2017; **4**.

7. Salum GA, Gadelha A, Pan PM, Moriyama TS, Graeff-Martins AS, Tamanaha AC *et al.* High risk cohort study for psychiatric disorders in childhood: rationale, design, methods and preliminary results. *International Journal of Methods in Psychiatric Research* 2015; **24**(1)**:** 58-73.

8. Gold AL, Steuber ER, White LK, Pacheco J, Sachs JF, Pagliaccio D *et al.* Cortical thickness and subcortical gray matter volume in pediatric anxiety disorders. *Neuropsychopharmacology* 2017; **42**(12)**:** 2423-2433.

9. Gold AL, Abend R, Britton JC, Behrens B, Farber MJ, Ronkin EG *et al.* Age differences in the neural correlates of anxiety disorders: An fMRI study of response to learned threat. *American Journal of Psychiatry* 2020; **177:** 454-463.

10. Volzke H, Alte D, Schmidt CO, Radke D, Lorbeer R, Friedrich N *et al.* Cohort profile: The study of health in pomerania. *Int J Epidemiol* 2011; **40**(2)**:** 294-307.

11. Hamilton M. The assessment of anxiety-states by rating. *Br J Med Psychol* 1959; **32**(1)**:** 50-55.

12. Meyer TJ, Miller ML, Metzger RL, Borkovec TD. Development and validation of the Penn State Worry Questionnaire. *Behaviour Research and Therapy* 1990; **28**(6)**:** 487-495.

13. Spitzer RL, Kroenke K, Williams JBW, Lowe B. A brief measure for assessing generalized anxiety disorder - The GAD-7. *Archives of Internal Medicine* 2006; **166**(10)**:** 1092-1097.

14. Spielberger CD, Gorsuch RL, Lushene R, Vagg PR, Jacobs GA. *Manual for the State-Trait Anxiety Inventory*. Consulting Psychologists Press: Palo Alto, CA, 1983.

15. Taylor S, Zvolensky MJ, Cox BJ, Deacon B, Heimberg RG, Ledley DR *et al.* Robust dimensions of anxiety sensitivity: Development and initial validation of the anxiety sensitivity index-3. *Psychological Assessment* 2007; **19**(2)**:** 176-188.

16. Beck AT, Epstein N, Brown G, Steer RA. An inventory for measuring clinical anxiety: psychometric properties. *J Consult Clin Psychol* 1988; **56**(6)**:** 893-897.

17. Heimberg RG, Horner KJ, Juster HR, Safren SA, Brown EJ, Schneier FR *et al.* Psychometric properties of the Liebowitz Social Anxiety Scale. *Psychological Medicine* 1999; **29**(1)**:** 199-212.

18. Bandelow B. Assessing the efficacy of treatments for panic disorder and agoraphobia: II. The Panic and Agoraphobia Scale. *International Clinical Psychopharmacology* 1995; **10**(2)**:** 73-81.

19. Chambless DL, Caputo GC, Bright P, Gallagher R. Assessment of fear of fear in agoraphobics - The body sensations questionnaire and the agoraphobic cognitions questionnaire. *J Consult Clin Psychol* 1984; **52**(6)**:** 1090-1097.

20. Shear MK, Rucci P, Williams J, Frank E, Grochocinski V, Vander Bilt J *et al.* Reliability and validity of the Panic Disorder Severity Scale: replication and extension. *Journal of Psychiatric Research* 2001; **35**(5)**:** 293-296.

21. Beck AT, Steer RA, K. BG. *Beck Depression Inventory-II*. The Psychological Corporation: San Antonio, TX, 1996.

22. Hamilton M. A rating scale for depression. *J Neurol Neurosurg Psychiatry* 1960; **23**(1)**:** 56-62.

23. Birmaher B, Khetarpal S, Brent D, Cully M, Balach L, Kaufman J *et al.* The screen for child anxiety related emotional disorders (SCARED): Scale construction and psychometric characteristics. *J Am Acad Child Adolesc Psychiatr* 1997; **36**(4)**:** 545-553.

24. Saylor CF, Finch A, Spirito A, Bennett B. The Children's Depression Inventory: A systematic evaluation of psychometric properties. *J Consult Clin Psychol* 1984; **52**(6)**:** 955-967.

25. Barch DM, Albaugh MD, Avenevoli S, Chang L, Clark DB, Glantz MD *et al.* Demographic, physical and mental health assessments in the adolescent brain and cognitive development study: Rationale and description. *Developmental Cognitive Neuroscience* 2018; **32:** 55-66.

26. Wechsler D, Coalson DL, Raiford SE. *WAIS-IV technical and interpretive manual*. Pearson: San Antonio, TX, 2008.

27. Wechsler D. *Manual for the Wechsler Intelligence Scale for Children - Third Edition (WISC-III)*. The Psychological Corporation: San Antonio, TX, 1991.

28. Wechsler D. *Wechsler Abbreviated Scale of Intelligence*. The Psychological Corporation: San Antonio, TX, 1999.

29. Schoenberg MR, Scott JG, Duff K, Adams RL. Estimation of WAIS-III intelligence from combined performance and demographic variables: Development of the OPIE-3. *Clinical Neuropsychologist* 2002; **16**(4)**:** 426-438.

30. Bartz WR, Loy DL. The Shipley-Hartford as a brief IQ screening device. *Journal of Clinical Psychology* 1970.

31. Zachary RA, Shipley WC. *Shipley institute of living scale: Revised manual*. WPS, Western Psychological Services1986.

32. Zhang YY, Brady M, Smith S. Segmentation of brain MR images through a hidden Markov random field model and the expectation-maximization algorithm. *IEEE Trans Med Imaging* 2001; **20**(1)**:** 45-57.

33. Norbom LB, Doan NT, Alnaes D, Kaufmann T, Moberget T, Rokicki J *et al.* Probing Brain Developmental Patterns of Myelination and Associations With Psychopathology in Youths Using Gray/White Matter Contrast. *Biological Psychiatry* 2019; **85**(5)**:** 389-398.

34. Fischl B, Salat DH, Busa E, Albert M, Dieterich M, Haselgrove C *et al.* Whole brain segmentation: Automated labeling of neuroanatomical structures in the human brain. *Neuron* 2002; **33**(3)**:** 341-355.
