# Supplementary material for: Cortical and subcortical brain structure in generalized anxiety disorder: findings from 28 research sites in the ENIGMA-Anxiety Working Group
Source: Transl Psychiatry. 2021 Oct 1;11:502. doi: 10.1038/s41398-021-01622-1 (PMC8486763; doi:10.1038/s41398-021-01622-1)
Supplement: Supplementary file 2 — Supplemental Tables [file 41398_2021_1622_MOESM2_ESM.docx]

Supplementary Table 1. Descriptive statistics for sex, age, IQ and years of education for all sites in the secondary analysis with fixed slopes and random intercepts.

| **Site** | **Total** | **Healthy controls** | | | | | | | | | | **Individuals with GAD** | | | | | | | | | | | |
| --- | --- | --- | --- | --- | --- | --- | --- | --- | --- | --- | --- | --- | --- | --- | --- | --- | --- | --- | --- | --- | --- | --- | --- |
|  | **n** | **n** | **% F** | **Min age** | **Max age** | **Mean age** | **SD**  **age** | **Mean IQ** | **SD**  **IQ** | **Mean Edu** | **SD**  **Edu** | **Current GAD** | **% F** | **LT GAD** | **% F** | **Min age** | **Max age** | **Mean age** | **SD**  **age** | **Mean**  **IQ** | **SD**  **IQ** | **Mean Edu** | **SD**  **Edu** |
| ABCD | 1451 | 1347 | 53.8 | 8.9 | 11.0 | 10.00 | 0.62 |  |  | 4.37 | 0.76 | 20 | 60.0 | 84 | 56.0 | 9.0 | 10.9 | 10.12 | 0.66 |  |  | 4.34 | 0.80 |
| Barcelona | 89 | 58 | 69.0 | 18.0 | 40.0 | 22.07 | 4.37 |  |  | 14.45 | 2.14 | 30 | 66.7 | 1 | 100.0 | 18.0 | 33.0 | 23.13 | 4.57 |  |  | 14.61 | 2.22 |
| Baylor | 228 | 130 | 50.8 | 12.0 | 79.0 | 24.26 | 12.84 |  |  |  |  | 98 | 41.8 | 0 | -- | 18.0 | 56.0 | 28.85* | 9.23 |  |  |  |  |
| BHRCS | 388 | 373 | 45.6 | 5.0 | 14.0 | 9.66 | 1.84 | 102.58 | 16.52 | 4.19 | 1.69 | 15 | 66.7 | 0 | -- | 7.0 | 11.0 | 9.87 | 1.46 | 102.60 | 23.00 | 4.00 | 1.25 |
| Boystown | 93 | 44 | 52.3 | 13.0 | 18.0 | 15.39 | 1.62 | 105.23 | 10.23 |  |  | 49 | 59.2 | 0 | -- | 13.0 | 18.0 | 15.80 | 1.37 | 102.53 | 13.47 |  |  |
| Chicago-Milad | 43 | 16 | 25.0 | 18.0 | 59.0 | 32.38 | 13.39 |  |  | 16.44 | 2.58 | 27 | 66.7 | 0 | -- | 19.0 | 51.0 | 30.26 | 9.90 |  |  | 16.52 | 2.31 |
| Chicago-Phan | 120 | 42 | 54.8 | 18.0 | 60.0 | 25.21 | 9.77 |  |  | 15.79 | 3.06 | 78 | 73.1 | 0 | -- | 18.0 | 58.0 | 26.90 | 8.69 |  |  | 16.26 | 3.13 |
| Cincinnati | 3 | 0 | -- |  |  |  |  |  |  |  |  | 3 | 66.7 | 0 | -- | 12.0 | 16.0 | 13.33 | 2.31 | 109.67 | 10.79 | 8.67 | 2.08 |
| CMI-HBN | 93 | 54 | 51.9 | 6.2 | 19.3 | 10.18 | 2.85 | 107.00 | 15.88 | 6.11 | 2.84 | 39 | 64.1 | 0 | -- | 6.9 | 21.7 | 13.27* | 3.61 | 104.62 | 15.62 | 9.10* | 3.51 |
| Dresden | 94 | 47 | 68.1 | 19.0 | 50.0 | 28.89 | 8.40 |  |  | 12.13 | 0.90 | 47 | 76.6 | 0 | -- | 18.0 | 51.0 | 30.23 | 10.05 |  |  | 12.04 | 1.04 |
| Duke | 23 | 12 | 58.3 | 6.0 | 10.0 | 7.83 | 1.03 | 104.00 | 12.86 |  |  | 6 | 66.7 | 5 | 100.0 | 6.0 | 8.0 | 6.73* | 0.65 | 105.45 | 13.00 |  |  |
| Harvard | 163 | 41 | 63.4 | 18.0 | 33.0 | 25.46 | 3.92 | 114.15 | 7.91 | 16.59 | 1.95 | 122 | 73.0 | 0 | -- | 18.0 | 36.0 | 24.89 | 4.25 | 112.88 | 11.82 | 16.07 | 2.34 |
| Houston | 190 | 184 | 62.5 | 8.1 | 68.2 | 32.82 | 14.33 |  |  | 14.96 | 4.05 | 6 | 66.7 | 0 | -- | 10.3 | 44.2 | 20.08* | 12.48 |  |  | 9.83* | 4.12 |
| IOL | 40 | 16 | 81.3 | 22.0 | 63.0 | 39.25 | 15.04 | 103.44 | 8.10 |  |  | 24 | 75.0 | 0 | -- | 21.0 | 64.0 | 41.29 | 13.34 | 101.21 | 7.48 |  |  |
| Milan | 67 | 38 | 52.6 | 21.0 | 64.0 | 36.05 | 12.85 | 125.39 | 5.69 | 12.28 | 4.42 | 29 | 58.6 | 0 | -- | 20.9 | 69.9 | 43.18* | 14.54 | 120.21* | 10.41 | 10.80 | 4.24 |
| Muenster | 53 | 29 | 58.6 | 19.0 | 55.0 | 27.83 | 9.20 |  |  | 12.90 | 0.49 | 24 | 75.0 | 0 | -- | 20.0 | 56.0 | 29.21 | 10.34 |  |  | 12.46 | 1.74 |
| Pittsburgh-Andreescu | 59 | 32 | 56.3 | 19.0 | 90.0 | 57.06 | 18.04 |  |  | 15.88 | 3.79 | 27 | 77.8 | 0 | -- | 23.0 | 79.0 | 54.04 | 18.22 |  |  | 15.56 | 2.49 |
| Pittsburgh-Price | 55 | 0 | -- |  |  |  |  |  |  |  |  | 55 | 78.2 | 0 | -- | 18.0 | 54.0 | 30.33 | 10.15 |  |  | 14.45 | 2.40 |
| PROTAIA | 14 | 10 | 60.0 | 14.0 | 22.0 | 17.70 | 2.50 |  |  | 12.70 | 2.26 | 4 | 75.0 | 0 | -- | 14.0 | 18.0 | 16.75 | 1.89 |  |  | 11.50 | 1.29 |
| SanRaffaele | 91 | 70 | 80.0 | 23.0 | 63.0 | 45.50 | 10.88 |  |  | 15.56 | 3.22 | 21 | 66.7 | 0 | -- | 24.0 | 61.0 | 44.19 | 10.82 |  |  | 11.81* | 2.71 |
| SDAN | 367 | 219 | 57.1 | 8.4 | 47.2 | 17.07 | 6.43 | 114.09 | 11.85 |  |  | 129 | 64.3 | 19 | 73.7 | 8.1 | 50.3 | 16.11 | 8.61 | 113.53 | 13.70 |  |  |
| SHIP | 34 | 24 | 75.0 | 41.0 | 70.0 | 57.17 | 8.25 |  |  | 10.13 | 1.55 | 0 | -- | 10 | 70.0 | 41.0 | 67.0 | 55.40 | 8.09 |  |  | 10.45 | 1.54 |
| SNFA | 24 | 19 | 57.9 | 21.0 | 45.0 | 28.37 | 7.78 | 121.00 | 11.78 |  |  | 5 | 80.0 | 0 | -- | 19.0 | 31.0 | 24.00 | 5.66 | 123.00 | 4.95 |  |  |
| StonyBrook | 60 | 20 | 100 | 19.0 | 44.0 | 21.50 | 5.75 |  |  |  |  | 40 | 100 | 0 | -- | 18.0 | 49.0 | 22.98 | 6.04 |  |  |  |  |
| Sussex | 40 | 21 | 85.7 | 19.0 | 55.0 | 28.67 | 9.45 |  |  | 12.14 | 2.57 | 19 | 89.5 | 0 | -- | 18.0 | 44.0 | 29.79 | 7.00 |  |  | 12.89 | 1.82 |
| UCSD | 84 | 39 | 66.7 | 18.0 | 52.0 | 23.67 | 8.17 |  |  | 13.92 | 2.06 | 45 | 75.6 | 0 | -- | 18.0 | 53.0 | 29.09* | 11.22 |  |  | 14.51 | 2.29 |
| UPenn | 381 | 370 | 50.0 | 8.0 | 23.0 | 14.84 | 3.93 | 105.73 | 16.19 | 8.19 | 3.79 | 0 | -- | 11 | 63.6 | 8.0 | 19.0 | 16.36 | 3.32 | 107.45 | 18.09 | 9.91 | 3.36 |
| WashU | 47 | 27 | 48.1 | 8.0 | 12.0 | 9.85 | 1.06 |  |  |  |  | 20 | 65.0 | 0 | -- | 8.0 | 12.0 | 10.00 | 1.56 |  |  |  |  |
| **Total** | **4394** | **3282** | **55.0** | **5.0** | **90.0** | **16.51** | **11.94** | **107.44** | **15.74** | **7.22** | **4.78** | **982** | **68.4** | **130** | **62.3** | **6.0** | **79.0** | **24.23*** | **13.21** | **110.57*** | **14.29** | **12.38*** | **4.82** |

^* Indicates a significant difference between individuals with GAD and healthy controls^

^Note: GAD = Generalized anxiety disorder; F = female; Min = minimum; Max = maximum; SD = standard deviation; IQ = intelligence quotient; Edu = education in years; LT = life-time; ABCD = Adolescent Brain Cognitive Development^

^Study; BHRCS = Brazilian High Risk Cohort Study; CMI-HBN = Child Mind Institute Healthy Brain Network; IOL = Institute of Living; PROTAIA = Anxiety Disorders Program for^

^Child and Adolescent Psychiatry; SDAN = Section on Development and Affective Neuroscience; SHIP = Study of Health in Pomerania; SNFA = Section on Neurobiology of Fear and Anxiety; UCSD = University of California – San Diego; UPenn = University of Pennsylvania; WashU = Washington University.^

#

#

Supplementary Table 2. Other diagnoses for all sites in the secondary analysis with fixed slopes and random intercepts.

| **Site** | **SAD** | | **PD** | | **AG** | | **SPH** | | **Other ANXD** | | **MDD** | | **OCD** | | **PTSD** | | **SUD** | | **OtherD** | |
| --- | --- | --- | --- | --- | --- | --- | --- | --- | --- | --- | --- | --- | --- | --- | --- | --- | --- | --- | --- | --- |
|  | **Cur** | **LT** | **Cur** | **LT** | **Cur** | **LT** | **Cur** | **LT** | **Cur** | **LT** | **Cur** | **LT** | **Cur** | **LT** | **Cur** | **LT** | **Cur** | **LT** | **Cur** | **LT** |
| ABCD | 4 | 16 | 0 | 3 | 0 | 1 | 21 | 30 | 4 | 45 | 3 | 10 | 18 | 5 | 0 | 8 | 1 | 0 | 61 | 28 |
| Barcelona | 3 | 0 | 0 | 0 | 0 | 0 | 1 | 0 | 0 | 0 | 0 | 2 | 0 | 0 | 0 | 0 | 0 | 0 | 1 | 1 |
| Baylor | 26 | 0 | 3 | 2 | 2 | 1 | 1 | 1 | 0 | 0 | 62 | 1 | 5 | 3 | 14 | 2 | 50 | 7 | 80 | 1 |
| BHRCS | 1 | 0 | 0 | 0 | 0 | 0 | 2 | 0 | 1 | 4 | 4 | 0 | 0 | 0 | 0 | 0 | *NA* | *NA* | 5 | 0 |
| Boystown | 30 | 0 | *NA* | *NA* | *NA* | *NA* | *NA* | *NA* | 0 | 0 | 19 | 0 | *NA* | *NA* | 16 | 0 | *NA* | *NA* | 0 | 0 |
| Chicago-Milad | 5 | 0 | 5 | 0 | 2 | 0 | 6 | 0 | *NA* | *NA* | *NA* | *NA* | *NA* | *NA* | *NA* | *NA* | *NA* | *NA* | *NA* | *NA* |
| Chicago-Phan | 50 | 0 | 21 | 0 | 6 | 0 | 14 | 0 | 0 | 0 | 42 | 1 | 2 | 0 | 14 | 2 | 1 | 0 | 3 | 0 |
| Cincinnati | 1 | 0 | 0 | 0 | 0 | 0 | 0 | 0 | 0 | 0 | 0 | 0 | 0 | 0 | 0 | 0 | 0 | 0 | 3 | 0 |
| CMI-HBN | 9 | 0 | 0 | 0 | 0 | 0 | 6 | 0 | 5 | 0 | 14 | 0 | 0 | 0 | 0 | 0 | 3 | 0 | *NA* | *NA* |
| Dresden | 6 | 0 | 7 | 3 | 7 | 1 | 11 | 0 | 2 | 1 | 12 | 6 | 0 | 0 | 0 | 2 | 0 | 0 | 13 | 5 |
| Duke | 1 | 1 | *NA* | *NA* | *NA* | *NA* | *NA* | *NA* | 2 | 1 | *NA* | *NA* | *NA* | *NA* | *NA* | *NA* | *NA* | *NA* | *NA* | *NA* |
| Harvard | 75 | 2 | 17 | 12 | 14 | 3 | 25 | 2 | *NA* | *NA* | 29 | 64 | 14 | 4 | 10 | 6 | 0 | 5 | 13 | 8 |
| Houston | 2 | 0 | 1 | 0 | 0 | 0 | 0 | 0 | *NA* | *NA* | 6 | 0 | 1 | 0 | 0 | 0 | 1 | 0 | *NA* | *NA* |
| IOL | 6 | *NA* | 2 | *NA* | 1 | *NA* | 0 | *NA* | 0 | *NA* | 9 | *NA* | 0 | *NA* | 0 | *NA* | 0 | *NA* | 2 | *NA* |
| Milan | 0 | 0 | 0 | 5 | 0 | 0 | 0 | 0 | 2 | 1 | 0 | 8 | 0 | 0 | 0 | 0 | 0 | 1 | 0 | 2 |
| Muenster | *NA* | *NA* | *NA* | *NA* | *NA* | *NA* | *NA* | *NA* | *NA* | *NA* | *NA* | *NA* | *NA* | *NA* | *NA* | *NA* | *NA* | *NA* | *NA* | *NA* |
| Pittsburgh-Andreescu | 2 | 0 | 4 | 1 | 0 | 0 | 0 | 0 | *NA* | *NA* | 0 | 5 | 0 | 0 | 1 | 0 | 1 | 0 | 2 | 4 |
| Pittsburgh-Price | 17 | 0 | 8 | 8 | 7 | 0 | 5 | 0 | 0 | 0 | 14 | 0 | 4 | 0 | 7 | 0 | 0 | 0 | 6 | 0 |
| PROTAIA | 1 | 0 | 0 | 0 | 0 | 0 | 1 | 0 | 1 | 0 | 2 | 0 | 0 | 0 | 0 | 0 | 0 | 0 | 1 | 0 |
| SanRaffaele | 0 | 0 | 2 | 0 | 2 | 0 | *NA* | *NA* | *NA* | *NA* | 21 | 0 | *NA* | *NA* | *NA* | *NA* | *NA* | *NA* | 0 | 0 |
| SDAN | 80 | 12 | 2 | 0 | 0 | 0 | 46 | 0 | 0 (*23 NA)* | 0 (*23 NA)* | 2 | 2 | 0 | 0 | 0 | 0 | 0 | 0 | 6 (*23 NA)* | 1 (*23 NA)* |
| SHIP | 0 | 2 | 0 | 2 | 0 | 1 | 0 | 3 | 0 | 0 | 0 | 8 | 0 | 0 | 0 | 0 | 0 | 3 | 0 | 0 |
| SNFA | 3 | 0 | 0 | 0 | 0 | 0 | 0 | 0 | 0 | 0 | 0 | 0 | 0 | 0 | 0 | 0 | 0 | 0 | 0 | 0 |
| StonyBrook | 0 | 0 | 0 | 0 | 0 | 0 | 0 | 0 | 0 | 0 | 20 | 0 | 0 | 0 | 0 | 0 | 0 | 0 | 0 | 0 |
| Sussex | 0 | 0 | 0 | 0 | 0 | 0 | 0 | 0 | 0 | 0 | 0 | 0 | 0 | 0 | 0 | 0 | 0 | 0 | 0 | 0 |
| UCSD | 20 | 4 | 4 | 2 | 4 | 0 | *NA* | *NA* | 0 | 0 | 8 | 8 | 2 | 0 | 0 (*14 NA)* | 2 (*14 NA)* | 0 | 3 | 0 | 0 |
| UPenn | 0 | 4 | 0 | 1 | 0 | 1 | 0 | 5 | 0 | 4 | 0 | 7 | 0 | 2 | 0 | 4 | *NA* | *NA* | 0 | 8 |
| WashU | 9 | 0 | 0 | 0 | 0 | 0 | 0 | 1 | 3 | 1 | 3 | 0 | 0 | 0 | 0 | 0 | 0 | 0 | 3 | 6 |
| **Total** | **351** | **41** | **76** | **39** | **45** | **8** | **139** | **42** | **20** | **57** | **270** | **122** | **46** | **14** | **62** | **26** | **57** | **19** | **199** | **64** |

^Note: SAD = social anxiety disorder; PD = panic disorder; AG = agoraphobia; SPH = specific phobia; Other ANXD = other anxiety disorder; MDD = major depressive disorder; OCD = obsessive-compulsive disorder; PTSD = post-traumatic stress disorder; SUD = substance use dependence; OtherD = other disorder; Cur = current; LT = lifetime; NA = not available; ABCD = Adolescent Brain Cognitive Development Study; BHRCS = Brazilian High Risk Cohort Study; CMI-HBN = Child Mind Institute Healthy Brain Network; IOL = Institute of Living; PROTAIA = Anxiety Disorders Program for Child and Adolescent Psychiatry; SDAN = Section on Development and Affective Neuroscience; SHIP = Study of Health in Pomerania; SNFA = Section on Neurobiology of Fear and Anxiety; UCSD = University of California – San Diego; UPenn = University of Pennsylvania; WashU = Washington University^

#

#

Supplementary Table 3. Descriptive statistics for medication at scan for all sites in the secondary analysis with fixed slopes and random intercepts.

| **Site** | **Total** | **Healthy controls** | | | | | **Individuals with GAD** | | | | | |
| --- | --- | --- | --- | --- | --- | --- | --- | --- | --- | --- | --- | --- |
|  |  |  |  |  |  |  |  |  |  |  |  |  |
|  | **n** | **n** | **SSRI**  **SNRI** | **Benzo** | **Apsy** | **Other Med** | **Current GAD** | **LT GAD** | **SSRI**  **SNRI** | **Benzo** | **Apsy** | **Other Med** |
| ABCD | 1451 | 1347 | 1 | 0 | 0 | 94 | 20 | 84 | 8 | 1 | 1 | 25 |
| Barcelona | 90 | 59 | 0 | 0 | 0 | 0 | 30 | 1 | 0 | 1 | 0 | 0 |
| Baylor | 228 | 130 | 0 | 0 | 0 | 0 | 98 | 0 | 53 (23 *NA*) | 32 (23 *NA*) | 20 (23 *NA*) | 66 (23 *NA*) |
| BHRCS | 388 | 373 | 0 | *NA* | *NA* | 3 | 15 | 0 | 0 | *NA* | *NA* | 0 |
| Boystown | 94 | 45 | 0 | 0 | 0 | 1 | 49 | 0 | 16 | 1 | 13 | 11 |
| Chicago-Milad | 43 | 16 | *NA* | NA | *NA* | *NA* | 27 | 0 | *NA* | *NA* | *NA* | *NA* |
| Chicago-Phan | 120 | 42 | 0 | 0 | 0 | 0 | 78 | 0 | 0 | 0 | 0 | 0 |
| Cincinnati | 3 | 0 | -- | -- | -- | -- | 3 | 0 | 1 | 0 | 0 | 1 |
| CMI-HBN | 93 | 54 | 0 (30 *NA*) | *NA* | 0 (30 *NA*) | 4 (30 *NA*) | 39 | 0 | 2 (25 *NA*) | *NA* | 0 (25 *NA*) | 3 (25 *NA*) |
| Dresden | 94 | 47 | 0 | 0 | 0 | 0 | 47 | 0 | 0 | 0 | 0 | 1 |
| Duke | 23 | 12 | *NA* | *NA* | *NA* | *NA* | 6 | 5 | *NA* | *NA* | *NA* | *NA* |
| Harvard | 163 | 41 | 0 | 0 | 0 | 0 | 122 | 0 | 24 | 15 | 0 | 9 |
| Houston | 190 | 184 | 0 | 0 | 0 | 0 | 6 | 0 | 0 | 0 | 0 | 0 |
| IOL | 40 | 16 | 0 | 0 | 0 | 0 | 24 | 0 | 8 | 9 | 3 | 1 |
| Milan | 67 | 38 | 2 | 0 | 0 | 2 | 29 | 0 | 15 | 7 | 1 | 2 |
| Muenster | 53 | 29 | 0 | 0 | 0 | 0 | 24 | 0 | 8 | 0 | 0 | 2 |
| Pittsburgh-Andreescu | 59 | 32 | 0 | 0 | 0 | 0 | 27 | 0 | 0 | 0 | 0 | 0 |
| Pittsburgh-Price | 55 | 0 | -- | -- | -- | -- | 55 | 0 | 0 | 0 | 0 | 0 |
| PROTAIA | 14 | 10 | 0 | 0 | 0 | 1 | 4 | 0 | 0 | 0 | 0 | 0 |
| SanRaffaele | 91 | 70 | 0 | 0 | 0 | 0 | 21 | 0 | 13 (1 *NA*) | 19 | 11 (1 *NA*) | 14 (1 *NA*) |
| SDAN | 367 | 219 | 0 | 0 | 0 | 0 | 129 | 19 | 0 | 0 | 0 | 0 |
| SHIP | 34 | 24 | 1 | 0 | 0 | 5 | 0 | 10 | 4 | 0 | 0 | 2 |
| SNFA | 24 | 19 | 0 | 0 | 0 | 0 | 5 | 0 | 0 | 0 | 0 | 0 |
| StonyBrook | 60 | 20 | 0 | 0 | 0 | 0 | 40 | 0 | 0 | 0 | 0 | 0 |
| Sussex | 40 | 21 | 0 | 0 | 0 | 0 | 19 | 0 | 3 | 0 | 0 | 1 |
| UCSD | 84 | 39 | 0 | 0 | 0 | 0 | 45 | 0 | 0 | 0 | 0 | 0 |
| UPenn | 381 | 370 | 4 | 1 | 0 | 12 | 0 | 11 | 3 | 1 | 2 | 2 |
| WashU | 47 | 27 | 0 | 0 | 0 | 0 | 20 | 0 | 0 | 0 | 0 | 0 |
| **Total** | **4394** | **3282** | **8** | **1** | **0** | **122** | **982** | **130** | **158** | **86** | **51** | **140** |

^Note: GAD = generalized anxiety disorder; LT = life-time; SSRI = selective serotonin reuptake inhibitor; SNRI = serotonin and norepinephrine reuptake inhibitors; Benzo = benzodiazepines; Apsy = antipsychotics; Other med = other psychotropic medication; NA = not available; ABCD = Adolescent Brain Cognitive Development Study; BHRCS = Brazilian High Risk Cohort Study; CMI-HBN = Child Mind Institute Healthy Brain Network; IOL = Institute of Living; PROTAIA = Anxiety Disorders Program for Child and Adolescent Psychiatry; SDAN = Section on Development and Affective Neuroscience; SHIP = Study of Health in Pomerania; SNFA = Section on Neurobiology of Fear and Anxiety; UCSD = University of California – San Diego; UPenn = University of Pennsylvania; WashU = Washington University^

#

Supplementary Table 4. Overview of dependent and independent variables and contrasts of interest in the analysis.

| **Dependent variables** | **Independent variables** | |
| --- | --- | --- |
|  | *Model 1* | *Model 2* |
| **Subcortical volume (16 regions)** | **GAD** | **GAD** |
| **Cortical surface area (68 regions)** | **Age** | **Age** |
| **Cortical thickness (68 regions)** | **Sex** | **Sex** |
|  | **Age^2^** | **Age^2^** |
|  | **Sex*Age** | **Sex*Age** |
|  | **Sex*Age^2^** | **Sex*Age^2^** |
|  | **GAD*Age** | **GAD*Age** |
|  | **GAD*Sex** | **GAD*Sex** |
|  | **GAD*Age^2^** | **GAD*Age^2^** |
|  | **GAD*Sex*Age** | **GAD*Sex*Age** |
|  | **GAD*Sex*Age^2^** | **GAD*Sex*Age^2^** |
|  | **Scanner** | **Scanner** |
|  | IQ | IQ |
|  | Education | Education |
|  | Medication | Medication |
|  | Comorbid SAD, PD, AG, SPH, MDD, OCD, PTSD, SUD | Comorbid SAD, PD, AG, SPH, MDD, OCD, PTSD, SUD |
|  |  | Total surface area |
|  |  | Mean thickness |
|  |  | Total intracranial volume |
|  | *Contrasts of interest* | *Contrasts of interest* |
|  | 1. GAD>HC | 1. GAD>HC |
|  | 2. GAD<HC | 2. GAD<HC |
|  | 3. GAD*Sex (positive) | 3. GAD*Sex (positive) |
|  | 4. GAD*Sex (negative) | 4. GAD*Sex (negative) |
|  | 5. GAD*Age | 5. GAD*Age |
|  | 6. GAD*Sex*Age | 6. GAD*Sex*Age |

^Note: All sites provided information on the variables in bold text, the variables in normal text were only included if they were available for that site.^

^GAD = generalized anxiety disorder; SAD = social anxiety disorder; PD = panic disorder; AG = agoraphobia; SPH = specific phobia; MDD = major depressive disorder; OCD = obsessive-compulsive disorder; PTSD = post-traumatic stress disorder; SUD = substance use dependence; HC = healthy control.^

Supplementary Table 5. Overview of the independent variables included per site.

| **ABCD** | **Barcelona** | **Baylor** | **BHRCS** | **Boystown** | **Chicago-Milad** | **Chicago-Phan** | **CMI-HBN** |
| --- | --- | --- | --- | --- | --- | --- | --- |
| GAD | GAD | GAD | GAD | GAD | GAD | GAD | GAD |
| Age | Age | Age | Age | Age | Age | Age | Age |
| Sex | Sex | Sex | Sex | Sex | Sex | Sex | Sex |
| Age2 | Age2 | Age2 | Age2 | Age2 | Age2 | Age2 | Age2 |
| Sex*Age | Sex*Age | Sex*Age | Sex*Age | Sex*Age | Sex*Age | Sex*Age | Sex*Age |
| Sex*Age2 | Sex*Age2 | Sex*Age2 | Sex*Age2 | Sex*Age2 | Sex*Age2 | Sex*Age2 | Sex*Age2 |
| GAD*Age | GAD*Age | GAD*Age | GAD*Age | GAD*Age | GAD*Age | GAD*Age | GAD*Age |
| GAD*Sex | GAD*Sex | GAD*Sex | GAD*Sex | GAD*Sex | GAD*Sex | GAD*Sex | GAD*Sex |
| GAD*Age2 | GAD*Age2 | GAD*Age2 | GAD*Age2 | GAD*Age2 | GAD*Age2 | GAD*Age2 | GAD*Age2 |
| GAD*Sex*Age | GAD*Sex*Age | GAD*Sex*Age | GAD*Sex*Age | GAD*Sex*Age | GAD*Sex*Age | GAD*Sex*Age | GAD*Sex*Age |
| GAD*Sex*Age2 | GAD*Sex*Age2 | GAD*Sex*Age2 | GAD*Sex*Age2 | GAD*Sex*Age2 | GAD*Sex*Age2 | GAD*Sex*Age2 | GAD*Sex*Age2 |
|  |  |  | IQ | IQ |  |  | IQ |
| Education | Education |  | Education |  | Education | Education | Education |
| Medication | Medication | Medication |  | Medication |  |  | Medication |
| SAD | SAD | SAD | SAD | SAD | SAD | SAD | SAD |
| PD |  | PD |  |  | PD | PD |  |
| AG |  | AG |  |  | AG | AG |  |
| SPH | SPH | SPH | SPH |  | SPH | SPH | SPH |
| MDD | MDD | MDD | MDD | MDD |  | MDD | MDD |
| OCD |  | OCD |  |  |  | OCD |  |
| PTSD |  | PTSD |  | PTSD |  | PTSD |  |
| SUD |  | SUD |  |  |  | SUD | SUD |

Supplementary Table 5 (continued).

| **Dresden** | **Duke** | **Harvard** | **IOL** | **Milan** | **Muenster** | **Pittsburgh-Andreescu** |
| --- | --- | --- | --- | --- | --- | --- |
| GAD | GAD | GAD | GAD | GAD | GAD | GAD |
| Age | Age | Age | Age | Age | Age | Age |
| Sex | Sex | Sex | Sex | Sex | Sex | Sex |
| Age2 |  | Age2 | Age2 | Age2 | Age2 | Age2 |
| Sex*Age | Sex*Age | Sex*Age | Sex*Age | Sex*Age | Sex*Age | Sex*Age |
| Sex*Age2 |  | Sex*Age2 | Sex*Age2 | Sex*Age2 | Sex*Age2 | Sex*Age2 |
| GAD*Age | GAD*Age | GAD*Age | GAD*Age | GAD*Age | GAD*Age | GAD*Age |
| GAD*Sex | GAD*Sex | GAD*Sex | GAD*Sex | GAD*Sex | GAD*Sex | GAD*Sex |
| GAD*Age2 |  | GAD*Age2 | GAD*Age2 | GAD*Age2 | GAD*Age2 | GAD*Age2 |
| GAD*Sex*Age | GAD*Sex*Age | GAD*Sex*Age | GAD*Sex*Age | GAD*Sex*Age | GAD*Sex*Age | GAD*Sex*Age |
| GAD*Sex*Age2 |  | GAD*Sex*Age2 | GAD*Sex*Age2 | GAD*Sex*Age2 | GAD*Sex*Age2 | GAD*Sex*Age2 |
|  | IQ | IQ | IQ | IQ |  |  |
| Education |  | Education |  | Education | Education | Education |
| Medication |  | Medication | Medication | Medication | Medication |  |
| SAD | SAD | SAD | SAD |  |  | SAD |
| PD |  | PD | PD | PD |  | PD |
| AG |  | AG | AG |  |  |  |
| SPH |  | SPH |  |  |  |  |
| MDD |  | MDD | MDD | MDD |  | MDD |
|  |  | OCD |  |  |  |  |
| PTSD |  | PTSD |  |  |  | PTSD |
|  |  | SUD |  | SUD |  | SUD |

Supplementary Table 5 (continued).

| **SDAN** | **SHIP** | **StonyBrook** | **Sussex** | **UCSD** | **UPenn** | **WashU** |
| --- | --- | --- | --- | --- | --- | --- |
| GAD | GAD | GAD | GAD | GAD | GAD | GAD |
| Age | Age | Age | Age | Age | Age | Age |
| Sex | Sex |  | Sex | Sex | Sex | Sex |
| Age2 | Age2 | Age2 |  | Age2 | Age2 | Age2 |
| Sex*Age | Sex*Age |  | Sex*Age | Sex*Age | Sex*Age | Sex*Age |
| Sex*Age2 | Sex*Age2 |  |  | Sex*Age2 | Sex*Age2 | Sex*Age2 |
| GAD*Age | GAD*Age | GAD*Age | GAD*Age | GAD*Age | GAD*Age | GAD*Age |
| GAD*Sex | GAD*Sex |  | GAD*Sex | GAD*Sex | GAD*Sex | GAD*Sex |
| GAD*Age2 | GAD*Age2 | GAD*Age2 |  | GAD*Age2 | GAD*Age2 | GAD*Age2 |
| GAD*Sex*Age | GAD*Sex*Age |  | GAD*Sex*Age | GAD*Sex*Age | GAD*Sex*Age | GAD*Sex*Age |
| GAD*Sex*Age2 | GAD*Sex*Age2 |  |  | GAD*Sex*Age2 | GAD*Sex*Age2 | GAD*Sex*Age2 |
| IQ |  |  |  |  | IQ |  |
|  | Education |  | Education | Education | Education |  |
|  | Medication |  | Medication |  | Medication |  |
| SAD | AnyComorbid |  |  |  | AnyComorbid | SAD |
| PD |  |  |  |  |  |  |
|  |  |  |  |  |  |  |
| SPH |  |  |  |  |  | SPH |
| MDD |  | MDD |  |  |  | MDD |
|  |  |  |  |  |  |  |
|  |  |  |  |  |  |  |
|  |  |  |  |  |  |  |

^Note: GAD = generalized anxiety disorder; SAD = social anxiety disorder; PD = panic disorder; AG = agoraphobia; SPH = specific phobia; MDD = major depressive disorder; OCD = obsessive-compulsive disorder; PTSD = post-traumatic stress disorder; SUD = substance use dependence; ABCD = Adolescent Brain Cognitive Development Study; BHRCS = Brazilian High Risk Cohort Study; CMI-HBN = Child Mind Institute Healthy Brain Network; IOL = Institute of Living; SDAN = Section on Development and Affective Neuroscience; SHIP = Study of Health in Pomerania; UCSD = University of California – San Diego; UPenn = University of Pennsylvania; WashU = Washington University^
